# Cprp—An Unusual, Repetitive Protein Which Impacts Pleuromutilin Biosynthesis in the Basidiomycete *Clitopilus passeckerianus*

**DOI:** 10.3389/ffunb.2021.655323

**Published:** 2021-04-06

**Authors:** Kate M. J. de Mattos-Shipley, Gary D. Foster, Andy M. Bailey

**Affiliations:** School of Biological Sciences, University of Bristol, Bristol, United Kingdom

**Keywords:** pleuromutilin, secondary metabolism, CPRP, DDR48, *Clitopilus passeckerianus*, basidiomycete, antibiotic

## Abstract

Interrogation of an EST database for *Clitopilus passeckerianus* identified a putative homolog to the unusual stress response gene from yeast; *ddr48*, as being upregulated under pleuromutilin production conditions. Silencing of this gene, named *cprp*, produced a population of transformants which demonstrated significantly reduced pleuromutilin production. Attempts to complement a *Saccharomyces cerevisiae ddr48* mutant strain (strain Y16748) with *cprp* were hampered by the lack of a clearly identifiable mutant phenotype, but interestingly, overexpression of either *ddr48* or *cprp* in *S. cerevisiae* Y16748 led to a conspicuous and comparable reduction in growth rate. This observation, combined with the known role of DDR48 proteins from a range of fungal species in nutrient starvation and stress responses, raises the possibility that this family of proteins plays a role in triggering oligotrophic growth. Localization studies *via* the production of a Cprp:GFP fusion protein in *C. passeckerianus* showed clear localization adjacent to the hyphal septa and, to a lesser extent, cell walls, which is consistent with the identification of DDR48 as a cell wall-associated protein in various yeast species. To our knowledge this is the first study demonstrating that a DDR48-like protein plays a role in the regulation of a secondary metabolite, and represents the first DDR48-like protein from a basidiomycete. Potential homologs can be identified across much of the Dikarya, suggesting that this unusual protein may play a central role in regulating both primary and secondary metabolism in fungi.

## Introduction

**Figure 1:**
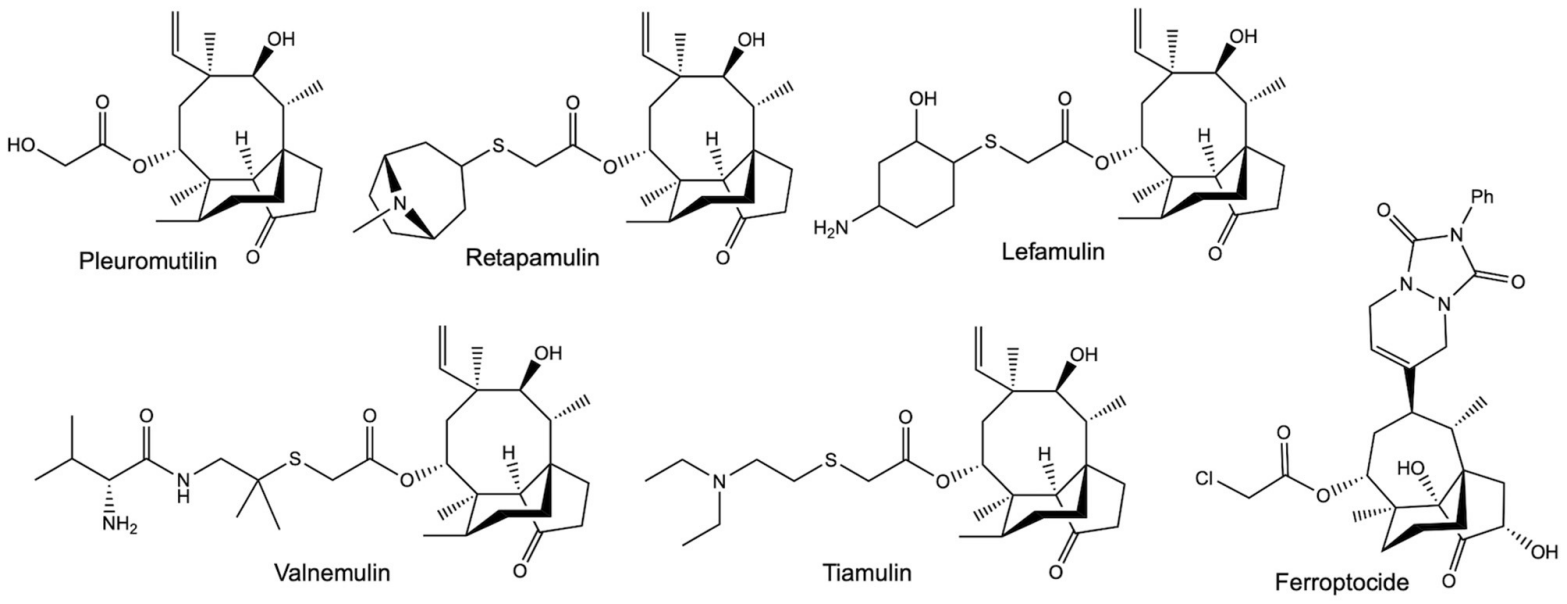
The structures of pleuromutilin and various pleuromutilin derivatives. Valnemulin and tiamulin are used in veterinary medicine as antibiotic agents. Retapamulin and Lefamulin both have approval for use in human medicine, as topical and oral / intravenous antibiotics, respectively. Ferroptocide is a recently produced antitumour derivative, which triggers ferroptosis *via* the inhibition of thioredoxin.

An increasingly valuable class of compounds for human therapeutics are the semi-synthetic pleuromutilin antibiotics (Figure 1). These compounds are all derived from a tricyclic diterpene natural product called pleuromutilin, which is produced by *Clitopilus passeckerianus* and related basidiomycete fungi (Hartley et al., 2009). Tiamulin and valnemulin (Figure 1) have been used in veterinary medicine for many years (Poulsen et al., 2001), but an increasing need for new antibiotics—largely due to the emergence of antibiotic resistant strains in community situations—has led to a surge in the development of pleuromutilin derivatives specifically for human use (Brooks et al., 2001; Bacqué et al., 2002; Springer et al., 2003; Hirokawa et al., 2009; Wang et al., 2012; Shang et al., 2013, 2014; Gao et al., 2017; Mu et al., 2017; Jin et al., 2019; Fan et al., 2020; Xie et al., 2020; Zhang et al., 2020). To date, two pleuromutilin antibiotics, retapamulin and lefamulin (Figure 1), have approval for use in human therapeutics (Butler, 2008; Lodise et al., 2020), and the development of further derivatives is ongoing, with the extended spectrum pleuromutilins (ESPs) showing promise against the difficult to treat Gram-negative bacteria (Paukner et al., 2013a,b, 2015a,b; Wicha et al., 2015; Thirring et al., 2017). There are also indications that pleuromutilins have the potential to be developed into much needed anti-tuberculosis agents (Lemieux et al., 2018).

The value of the pleuromutilins lies partly in their unique mode-of-action; inhibiting bacterial protein synthesis by selectively binding to conserved targets within the 50S ribosomal subunit (Hodgin and Högenauer, 1974, Schlünzen et al., 2004, Yan et al., 2006, Davidovich et al., 2007). This means that pleuromutilins are associated with low rates of resistance development and display minimal cross-resistance (Paukner and Riedl, 2017). Exciting recent work has also shown that pleuromutilin derivatives may have applications beyond their utility as antibiotic agents, with one highly derivatised compound named ferroptocide (Figure 1) exhibiting antitumour and immunomodulatory activities (Llabani et al., 2019).

The increasing importance of the pleuromutilins has naturally led to interest in both pleuromutilin biosynthesis and the biology of the producing species (de Mattos-Shipley et al., 2017). In the last 5 years, publications have reported the identification of the pleuromutilin biosynthetic gene cluster (BGC) from both *C. passeckerianus* (Bailey et al., 2016) and C. *pseudo-pinsitus* (Yamane et al., 2017), elucidation of the biosynthetic pathway *via* heterologous production of pleuromutilin, and the generation of novel derivatives *via* the biotransformation of semi-synthetic pathway intermediates (Alberti et al., 2017). What is currently poorly understood is the regulation of the biosynthetic pathway, particularly in terms of which genes and regulatory networks impact pleuromutilin production. Unlike many fungal natural products, the pleuromutilin BGC contains no pathway-specific regulatory genes (Bailey et al., 2016). It is possible that pathway specific transcription factors are located elsewhere in the genome, but it is also likely that biosynthesis is under the control of complex global regulatory networks, as seen in the regulation of gibberellin biosynthesis (Tudzynski, 2005). An understanding of pleuromutilin regulation could help inform strain improvement, which would in turn aid the development of the pleuromutilins as affordable mass market compounds, their expense currently being one of the major barriers to their widespread use (Watkins and File, 2020). Previous attempts at strain improvement have combined mutagenesis with media optimisation, but this has been met with limited success (Papa et al., 2006). In an attempt to gain insight into the regulation of pleuromutilin from a genetic perspective, an internal EST (Expressed Sequence Tag) database for *C. passeckerianus*, which was generated under differential pleuromutilin production and non-production conditions, was interrogated, and candidate genes of interest were identified.

We now report investigations into one such identified gene, which encodes an unusually repetitive protein we have named Cprp (*Clitopilus passeckerianus r*epetitive *p*rotein). Silencing of *cprp* has shown that it does indeed impact pleuromutilin biosynthesis, and localization studies have shown that Cprp accumulates adjacent to the septa and, to a lesser extent, the cell wall. A bioinformatic analysis has revealed that putative homologs, all containing a repetitive S-Y-G motif that may be involved in RNA binding, are frequent within the higher fungi, suggesting that they may play a conserved role across a range of fungal taxa.

## Results

### Initial Identification and Analysis of *cprp*

An internal EST database, containing libraries generated under pleuromutilin production and non-production conditions, was interrogated for candidate genes involved in the regulation of pleuromutilin. A requirement for differential expression was combined with specific search terms, based on knowledge concerning the environmental factors which impact pleuromutilin biosynthesis. One parameter used was homology to genes involved in nitrogen regulation, due to observed repression of pleuromutilin production upon the addition of sodium nitrate or glutamic acid to the culturing medium (Supplementary Figure 1). This identified EST contig c456 (accession number MW509766), which is upregulated during pleuromutilin production (eight representative EST sequences in the production library vs. one sequence in the non-production library) and has homology with *gsr1*, which encodes a putative nitrogen starvation response protein from *Fusarium fujikuroi*.

**Figure 2:**
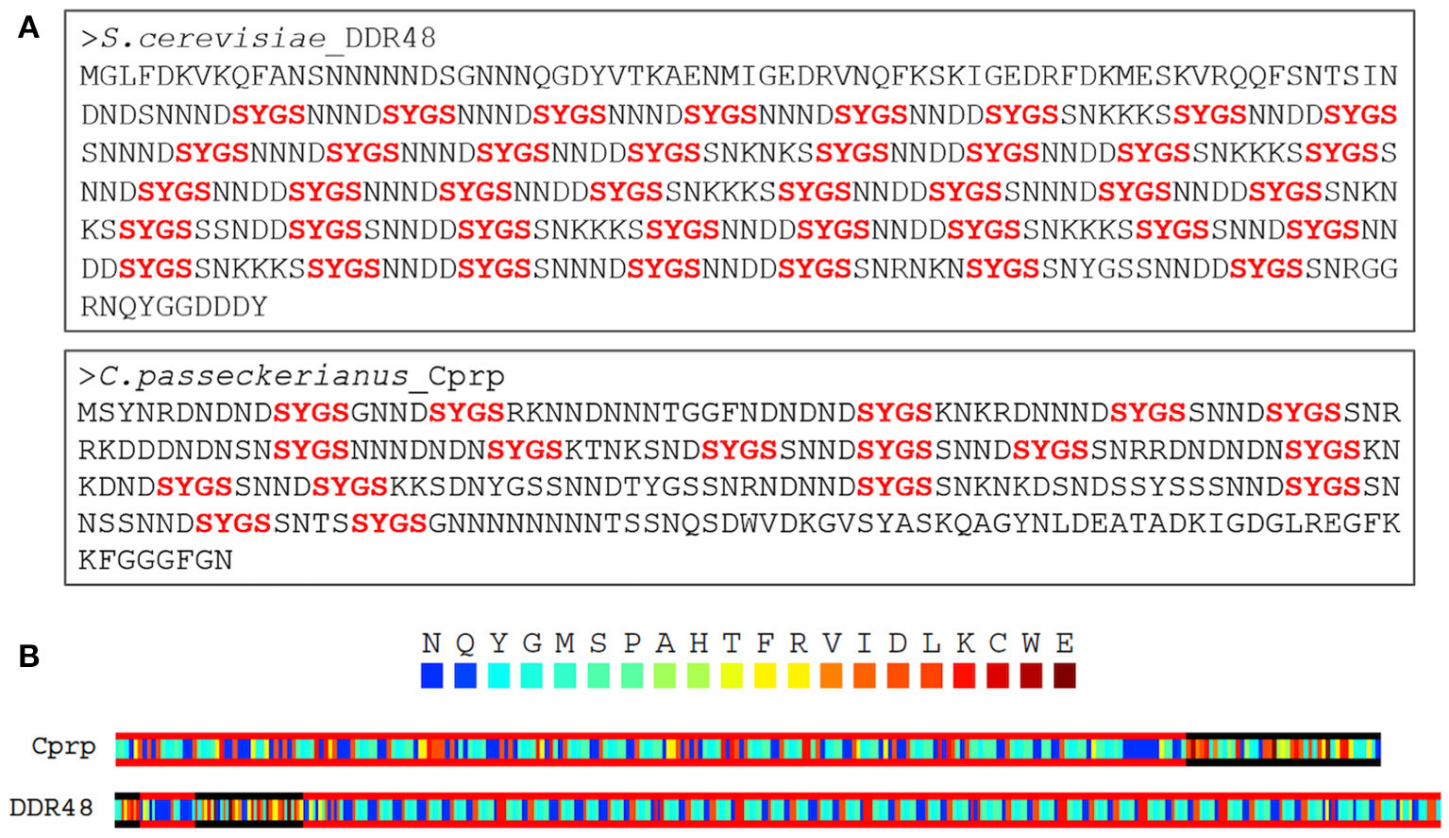
**(A)** Both DDR48 and Cprp are shown to be highly repetitive proteins with low amino acid sequence complexity, being enriched in asparagine, serine, aspartic acid, and glycine residues. Both contain multiple [G/S]-Y-[G/S] motifs (in red), which are known to be present in intrinsically disordered RNA-binding proteins. **(B)** A PLAAC analysis of Cprp and DDR48 showing regions (framed in red) which are predicted to be prion-like domains (Lancaster et al., 2014).

An alignment of the EST sequence with a genomic database for *C. passeckerianus* (Bailey et al., 2016) confirmed that c456 represents an entire coding sequence and allowed the identification of three introns, all with the GT-AG splice-site consensus. Translation to the predicted protein revealed a highly repetitive amino acid sequence, and thus the protein was named Cprp (*C. passeckerianus r*epetitive *p*rotein), with the encoding gene named *cprp*. Searching the NCBI Swiss-Prot protein database with Cprp determined that this protein shares significant sequence similarity (E = 3 × 10^−32^; 59.9 % protein identity) with the stress response protein DDR48 from *S. cerevisiae* (Accession number P18899.4; Supplementary Figure 2). Both DDR48 and Cprp are unusual proteins which are highly repetitive with low amino acid sequence complexity, being enriched in asparagine, serine, aspartic acid and glycine residues (Figure 2A). DDR48 consists largely of eight amino acid repeats with the sequence NNXDSYGS or similar. Cprp, although less consistently repetitive, contains multiple copies of a similar motif with the same core S-Y-G-S sequence (Figure 2A). Motifs containing S-Y-G are best known for their roles in RNA binding (Mitsuhashi et al., 2017), which could potentially indicate a role for these genes in RNA-mediated regulation. As RNA binding proteins are commonly composed of prion-like domains (PrLDs) (March et al., 2016), a PLAAC analysis, which scans protein sequences for domains with **p**rion-**l**ike **a**mino **a**cid **c**omposition was conducted (Lancaster et al., 2014). This determined that both DDR48 and Cprp are comprised largely of prion-like amino acid domains (Figure 2B; Supplementary Figures 3, 4). Finally, an NCBI conserved domain search (Lu et al., 2020) provided evidence that DDR48 and Cprp contain a PTZ00110 super family helicase domain (accession: cl36512; DDR48: E = 9.12 × 10^−04^; Cprp: E = 7.92 × 10^−04^).

### Silencing of *cprp* Reduces Antibiotic Production

**Figure 3:**
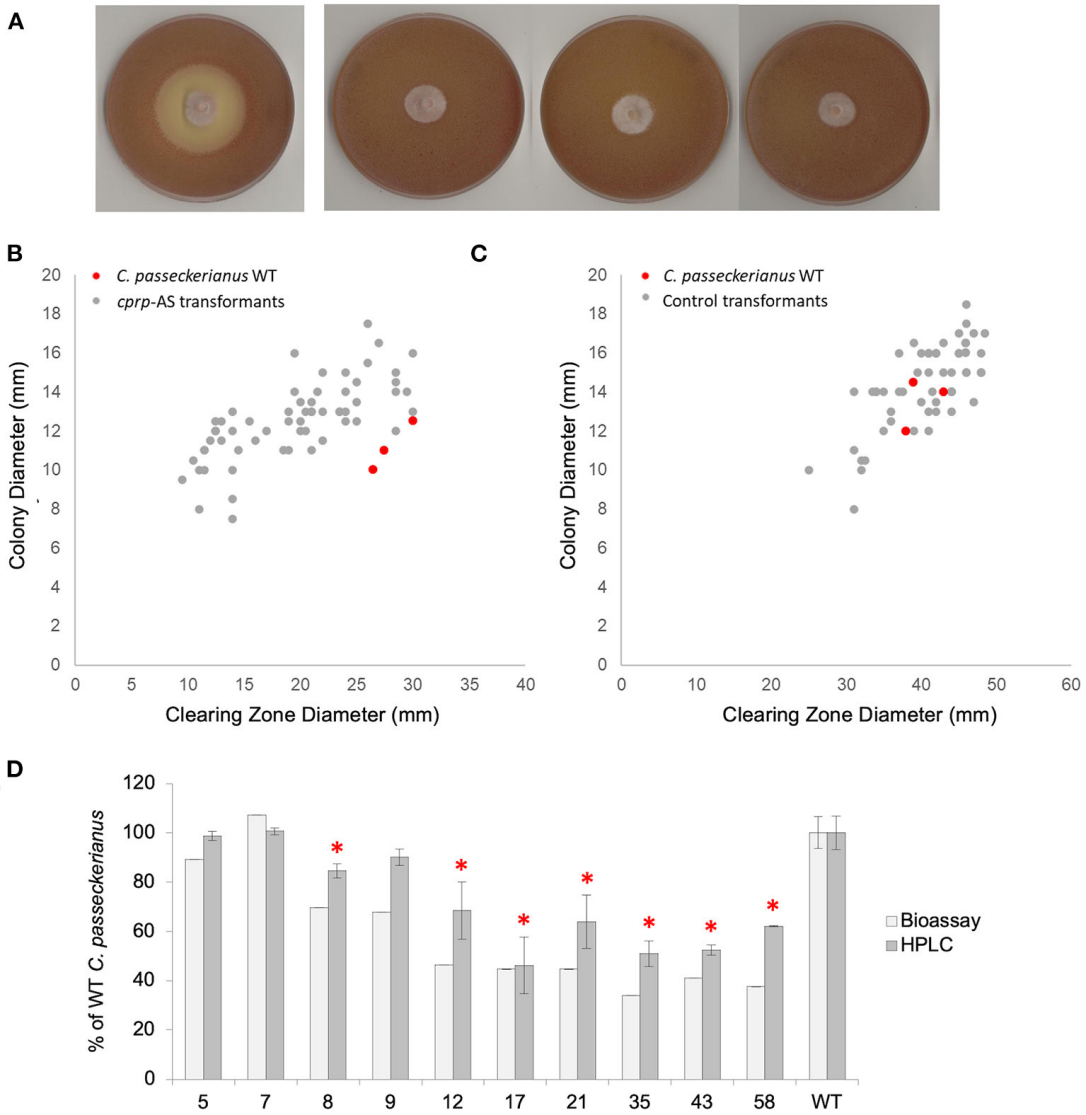
Analysis of pleuromutilin production in a population of *cprp*-AS (antisense) transformants. **(A)** Bioassay plates for wild-type *C. passeckerianus* and *cprp*-AS transformants 17, 35, and 58 (left to right). **(B)** Bioassay data for a population of 60 *cprp*-AS transformants. Full bioassay data for each transformant can be seen in Supplementary Figure 5. **(C)** Bioassay data for a population of 53 control transformants containing plasmid pYES-hph-CBXgene. Full bioassay data for each transformant can be seen in Supplementary Figure 5. **(D)** HPLC and plate-based analysis of pleuromutilin production for wild-type *C. passeckerianus* and selected *cprp*-AS transformants. Transformants 17, 35, and 58 produced no observable clearing zone beyond the edge of the colony; what is seen here is the colony diameter. Error bars represent the standard deviation of triplicate measurements. * denotes HPLC data which was found to be significantly different to the wild-type data (as determined by a two-tailed *t*-test. See Supplementary Table 1 for *P*-values).

To experimentally probe any relationship between Cprp and pleuromutilin biosynthesis a plasmid containing a *cprp* antisense (*cprp*-AS) cassette was constructed. Gene silencing using such an approach has previously been successfully employed for *C. passeckerianus* and aided the original identification of the pleuromutilin gene cluster (Bailey et al., 2016). Sixty resulting *C. passeckerianus cprp*-AS (antisense) transformants were subjected to an initial analysis of pleuromutilin production *via* plate-based bioassay (Figures 3A–C).

A clear reduction in *B. subtilis* inhibition, as measured by clearing zone diameter, was observed for many of the antisense transformants, with three transformants demonstrating no antibacterial activity at all (Figure 3A). The average clearing zone diameter was 70.0% of the wild type with the colony size ranging from 76.1 to 129% of the wild type with an average of 112.4% (Figure 3B; Supplementary Figure 5). A control set of transformants was generated using the original pYES-hph-CBXgene plasmid (Bailey et al., 2016) without the *cprp* antisense cassette. Although these control transformants demonstrated some variation in clearing zone diameter, any change could be accounted for by a comparable increase or decrease in growth rate, as demonstrated by a linear relationship between colony diameter and clearing zone diameter (Figure 3C; Supplementary Figure 5). Across the set of control transformants, the average colony diameter was 105.7% of wild-type and the average clearing zone was 100.8% of wild-type.

*Cprp*-AS transformants 5, 7, 8, 9, 12, 17, 21, 35, 43, and 58, which produced clearing zones of varying sizes, were subjected to further analysis. Diagnostic PCRs confirmed integration of the silencing cassette (Supplementary Figure 10) and northern blot analysis confirmed clear, albeit varying, silencing of *cprp* in all lines screened (Supplementary Figure 6). Fermentation in liquid production medium (CGC), chemical extraction and HPLC analysis confirmed that although all transformants produced some pleuromutilin, many demonstrated significantly reduced titres in liquid cultures, and these reductions were in agreement with the bioassay data (Figure 3D).

### Expression of *cprp* in an *S. cerevisiae ddr48* Mutant

To investigate whether Cprp is a true functional homolog of DDR48 from *S. cerevisiae*, complementation of a *ddr48* mutant strain (strain Y16758) with *cprp* was attempted. A series of plasmids were constructed to allow expression of *cprp* in strain Y16758. All constructs were built using the yeast expression vector pYES2-19, placing expression under the control of the strongly inducible pGAL1 promoter (West et al., 1984). All constructs were designed to include 20 bp of the 5′ UTR from *ddr48*, because in *S. cerevisiae*, this region of the 5′ UTR often contains a consensus sequence which improves the efficiency of transcription. This consensus sequence was then followed by either the *cprp* coding sequence (plasmid pYES2-CpcDNA) or the full gene including the three introns (plasmid pYES2-CpgDNA). The genomic construct was used as a negative control, to confirm that any phenotypes observed are due to the correct translation of Cprp. As a positive control, a construct was built containing the wild-type DDR48 gene (plasmid pYES2-DDR48). The three constructs were transformed into the *ddr48* mutant strain Y16758. In addition, pYES2-19 (lacking any insert) was transformed into both the *ddr48* mutant and the wild-type strain Y10000 as negative controls and to allow direct comparison with the various transformants on media lacking uracil.

The *ddr48* mutant Y16758 and parental strain Y10000 were subjected to a variety of examinations in an attempt to show a clear mutant phenotype, thus allowing any complementation to be assessed. A slight decrease in tolerance to heat-shock treatments had previously been reported in a *ddr48* mutant (Treger and McEntee, 1990), but unfortunately no difference could be detected between Y16758 and Y10000. *Ddr48* mutants were also reported to exhibit reduced spontaneous mutation rates, as measured by spontaneous resistance to canavanine or reversion to histidine prototrophy. Replicating the exact method used by Treger and McEntee (1990) failed to generate any canavanine resistance in either strain, even when 50 times the number of cells were plated out, and it was not possible to test mutation rates at the *his3* locus as the strains used in this work are *his3* deletion strains.

**Figure 4:**
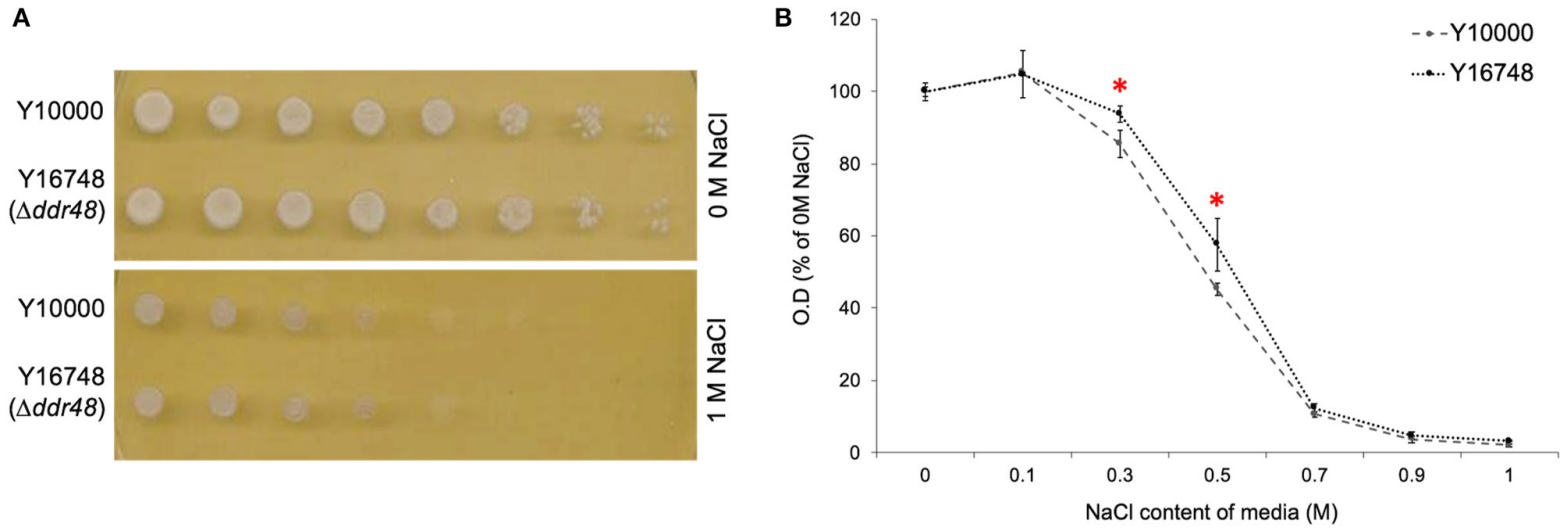
**(A)** Agar spotting assays for salt tolerance of *S. cerevisiae* strain Y10000 and Y16748 showed no apparent difference. **(B)** The optical density (O.D) of Y10000 and Y16748 (Δ*ddr48*) *S. cerevisiae* cultures 16 h after inoculation in media containing a range of NaCl concentrations. Error bars represent the standard deviation of triplicates. * Denotes NaCl concentrations where the responses of Y10000 and Y16748 (Δ*ddr48*) were found to be significantly different (as determined by a two-tailed *t*-test. See Supplementary Table 2 for *P*-values).

Another reported phenotype for *ddr48* mutants is an increased sensitivity to salt stress (Yoshikawa et al., 2009). In an attempt to reproduce this finding, agar spotting assays were conducted, but as is apparent in Figure 4A, there was no discernible difference in the growth of the wild-type strain (Y10000) or the *ddr48* mutant (Y16758). Growth rates were then measured in liquid cultures to more accurately assess any differences. A small but significant difference was observed at 0.3 and 0.5 M NaCl, with the *ddr48* mutant appearing to have a slightly higher tolerance to osmotic stress than the wild-type strain (Figure 4B), which directly contradicts the previous findings.

**Figure 5:**
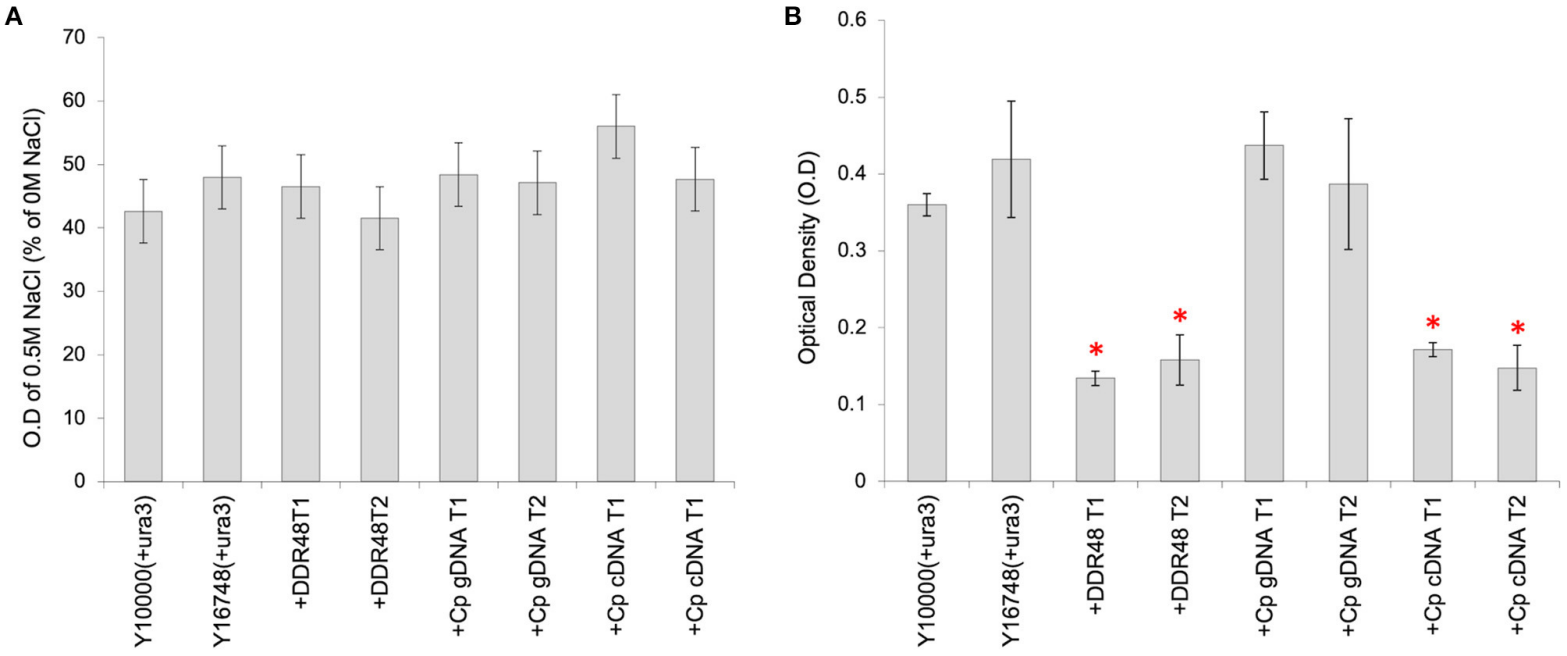
Analysis of *S. cerevisiae* strains Y10000 (“WT”) and Y16748 (Δ*ddr48*), as well as transformants highly expressing either DDR48 (+DDR48) or Cprp gDNA (+Cp gDNA) or cDNA (+Cp cDNA). Two separate transformants were assessed for each construct (T1 and T2). Error bars represent the standard deviation of triplicates. **(A)** Response to salt stress as measured by the O.D of cultures supplemented with 0.5 M NaCl, compared to cultures not supplemented. **(B)** Growth rate analysis, as measured by the O.D of cultures 16 h after inoculation. All cultures contained 20% (w/v) galactose to induce expression from the pGAL1 promoter. * Denotes transformants which had growth rates that were significantly different to strain Y10000 (+pYES2) (as determined by a two-tailed *t*-test. See Supplementary Table 3 for *P*-values).

Transformants containing the various *cprp* or *ddr48* expression constructs were subjected to similar screening in media supplemented with NaCl, but unfortunately no consistent pattern could be detected (Figure 5A). However, an unexpected consequence of overexpression of either DDR48 or Cprp was a clear and significant reduction in growth rate (Figure 5B). The average growth rate of mutant Y16748(+*ura3*) was slightly higher than the parental strain Y10000(+*ura3*), whereas the DDR48 overexpression transformants showed a striking decrease in growth rate when grown in cultures containing galactose (to induce expression), with an average optical density of only 37.2% of wild-type and 32.0% of the *ddr48* mutant. The yeast strains complemented with a *C. passeckerianus cprp* cDNA cassette showed a similar reduction in growth rate, being 44.3% of wild-type and 38.0% of the *ddr48* mutant (Figure 5B). Transformants containing a *cprp* cassette constructed using genomic DNA, which would not produce a functional protein in *S. cerevisiae*, did not show any reduction in growth rate, either in the presence or absence of galactose induction.

### Localization of Cprp in *C. passeckerianus*

**Figure 6:**
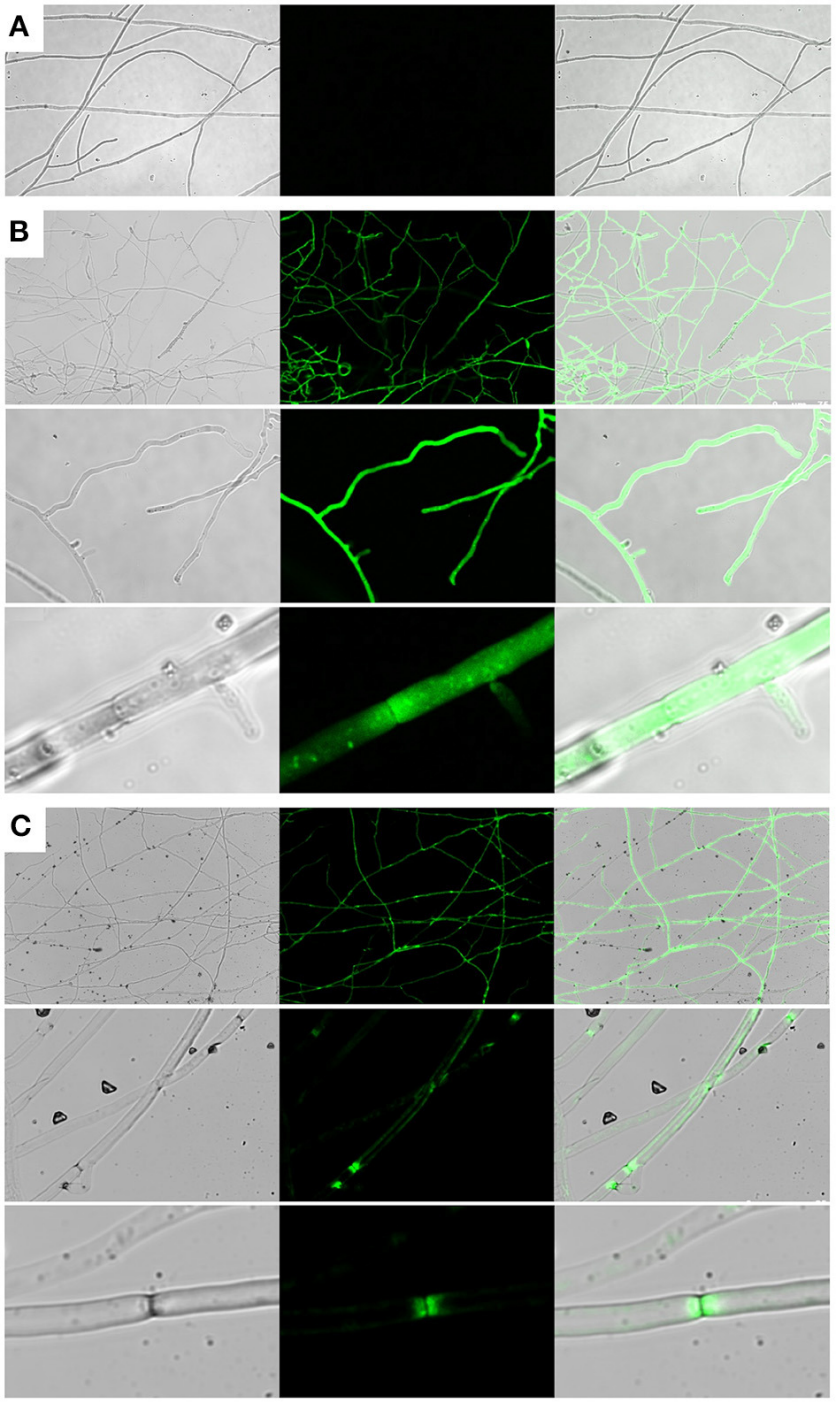
Cprp localization in *C. passeckerianus*. **(A)** Wild-type *C. passeckerianus*. **(B)** *C. passeckerianus* expressing GFP from plasmid p004iGM3, showing dispersal throughout the hyphae. **(C)** *C. passeckerianus* expressing a Cprp-GFP fusion protein, showing clear septal localization and increased fluorescence at the cell wall. Each image shows a phase contrast image, a fluorescent image and a merged image (left to right).

To investigate whether Cprp is localized to a particular subcellular region, a Cprp-GFP fusion protein was expressed in *C. passeckerianus*. A genomic fragment comprising the *cprp* promoter and coding region was fused with eGFP using yeast homologous recombination, replacing the stop codon of *cprp* with the start codon of GFP. The resulting plasmid, pYES-hph-cprpGFP, was transformed into *C. passeckerianus*, generating seven independent transformants. Two of these; Cprp-GFP-2 and Cprp-GFP-5, were found to fluoresce during initial screens so were prepared for confocal microscopy by growing mycelium across a sterilized microscope slide. A *C. passeckerianus* line expressing GFP under the control of P_gpd*II*_ on the plasmid p004iGM3 (Burns et al., 2005) was used as a negative control to confirm that GFP is not normally subject to localization in *C. passeckerianus*. Confocal microscopy confirmed that wild-type *C. passeckerianus* exhibits no detectable fluorescence (Figure 6A), whereas the strain expressing GFP from plasmid p004iGM3 exhibited cytoplasmic fluorescence with no particular localisation (Figure 6B). Confocal images of the Cprp-GFP transgenic lines showed clear evidence of localization, with fluorescence being concentrated adjacent to the septa and a slight increase in fluorescence at the periphery of the cell (Figure 6C).

### Bioinformatic Analysis of the DDR48/Cprp Family

The presence of a likely DDR48 homolog in *Clitopilus passeckerianus*, as well as the previously identified homologs in *Candida albicans* and *Histoplasma capsulatum* raises the question as to how widely distributed such proteins may be. To explore this, the NCBI nr protein database was searched for similar proteins (blastp algorithm; Expect threshold = 1 × 10^−05^) within the main fungal taxonomic divisions. Proteins demonstrating significant similarity to DDR48 and Cprp were identified within all subdivisions of the Basidiomycota (namely the Agaricomycotina, Pucciniomycotina, and Ustilaginomycotina) and within both the Saccharomycotina and Pezizomycotina ascomycete subdivisions (Table 1; Supplementary Table 4, Supplementary Figure 9). Interestingly, it was not possible to detect any putative homologs within the Taphrinomycotina, a monophyletic subdivision which is considered to be basal to the rest of the Ascomycota (Sugiyama et al., 2006). No proteins with significant sequence similarity with either Cprp or DDR48 from *S. cerevisiae* were identified within the Zygomycota, Chytridiomycota, Glomeromycota, or Neocallimastigomycota. One protein, accession number KNE54004.1, was identified within the Blastocladiomycotina and although this protein has relatively low similarity to DDR48 and Cprp (40.38 and 33.79% protein identity, respectively) it is similarly repetitive and contains a very similar motif (S-Y-D-S-Y-G-Y). An analysis of the predicted proteins found that although they vary quite significantly in length, they are all enriched in serine, asparagine, aspartic acid and glycine, and have a low negative GRAVY score (grand average of hydropathicity index), implying that they are largely non-polar and hydrophobic proteins (Supplementary Table 4). An analysis of the repetitive motifs in these proteins show that they all contain a core G/S-Y-G/S sequence, with S-Y-G being the dominant motif (See Supplementary Figure 9 for representative sequence logos).

**Table 1:** Representative DDR48-like proteins identified across the higher fungi.

**Species**	**Accession number**	**[G/S]Y[G/S] motif**				**PI with Cprp (%)**	**PI with DDR48 (%)**
		**SYG**	**SYS**	**GYS**	**GYG**		
**Ascomycota**							
**Pezizomycotina**							
*Fusarium fujikuroi*	XP_023428359.1	18	0	0	0	64.45	55.93
*Histoplasma capsulatum*	EER43993.1	16	1	0	0	42.54	56.16
*Talaromyces verruculosus*	KUL83446.1	26	0	0	0	68.50	72.77
*Colletotrichum tropicale*	KAF4827718.1	21	0	0	1	46.25	60.18
*Aspergillus oryzae*	EIT81464.1	23	0	0	1	51.16	55.00
**Saccharomycotina**
*Saccharomyces cerevisiae*	P18899.4	39	0	0	0	59.55	–
*Candida albicans*	XP_714253.1	19	0	0	0	75.27	67.06
*Zygotorulaspora mrakii*	QLG71076.1	45	0	0	0	60.00	65.95
*Naumovozyma dairenensis*	XP_003671904.1	35	1		0	52.38	66.39
**Basidiomycota**							
**Agaricomycotina**							
*Clitopilus passeckerianus*	–	17	1	0	0	–	59.55
*Coprinopsis cinerea*	XP_001835064.1	14	2	0	2	47.39	44.40
*Heterobasidion irregular*	XP_009552756.1	12	0	0	1	46.38	41.49
*Trametes versicolor*	XP_008040989.1	14	0	0	2	54.55	49.13
*Stereum hirsutum*	XP_007311260.1	14	0	0	1	52.24	44.20
**Ustilaginomycotina**							
*Testicularia cyperi*	PWY96941.1	20	0	0	0	64.00	56.62
*Violaceomyces palustris*	PWN49054.1	14	1	0	0	45.78	49.38
**Pucciniomycotina**							
*Microbotryum silenes-dioicae*	SGZ21624.1	6	1	0	2	32.56	40.96
**Blastocladiomycota**							
*Allomyces macrogynus*	KNE54004.1	15	0	0	13	33.79	40.38
*Homo sapiens* FUS	sp|P3563|	7	3	2	9	23.91	31.72
*Homo sapiens* TAF15	sp|Q92804|	4	4	3	23	31.85	33.50

*PI, Protein identity. See Supplementary Table 1 for additional sequences and further information regarding protein length, composition and GRAVY scores. These proteins share certain characteristics with a family of human RNA binding proteins, two of which (FUS and TAF15) are shown here*.

Although not identified through blast analysis, a literature search for any proteins with an S-Y-G motif rapidly identified a family of RNA-binding proteins from humans which are components of RNA granules and which are implicated in a spectrum of diseases (Mitsuhashi et al., 2017). These proteins are characterized by multiple copies of a (G/S-Y-G/S)-motif that has been identified as an RNA-binding site (Castello et al., 2016), are low-complexity (Kato et al., 2012) and are typically enriched in glutamine, and/or asparagine. Thus, although they have relatively low sequence similarity with DDR48 and Cprp, they share certain unusual characteristics. Three of these proteins; FUS, TAF15, and EWSR1 were included in this analysis and can be seen summarized in Table 1 and Supplementary Table 4. Their protein identities with Cprp and DDR48 ranged from 29.3 to 33.5%, and unlike the fungal proteins which contain almost exclusively S-Y-G motifs, the proteins from *Homo sapiens* include the full range of (G/S-Y-G/S) RNA binging motifs; namely S-Y-S, S-Y-G, G-Y-S, and G-Y-G (Supplementary Table 4, Supplementary Figure 9). Furthermore, the amino acids surrounding the core sequence vary far more, as can be seen in the sequence logos generated for these proteins (Supplementary Figure 9B).

Another feature that the fungal proteins share with the human RNA-binding proteins is that they are all predicted to be intrinsically disordered proteins (as analyzed by InterPro), which are characterized by a lack of defined three-dimensional structure.

## Discussion

To gain an insight into how pleuromutilin biosynthesis is regulated, a *C. passeckerianus* EST (Expressed Sequence Tag) database, containing libraries generated under production and non-production conditions, was searched for any sequences likely to impact pleuromutilin production. As pleuromutilin biosynthesis is repressed by the addition of nitrogenous compounds such as glutamic acid (Supplementary Figure 1), the libraries were specifically searched for sequences demonstrating similarity with genes involved in nitrogen regulation. Contig_456 (c456) was highlighted due to clear differential representation in pleuromutilin production and non-production libraries and sequence similarity to *gsr1* from *Fusarium fujikuroi*, a gene known to be strongly upregulated under nitrogen starvation conditions (Teichert et al., 2004, Teichert et al., 2006).

Annotation of the gene represented by EST c456 determined that it encodes a highly repetitive protein (named Cprp: *C. passeckerianus r*epetitive *p*rotein), with low sequence complexity and significant similarity (59.9% identity) with the stress-response protein DDR48. DDR48, originally identified in *S. cerevisiae*, has also been identified and investigated in *Candida albicans* and *Histoplasma capsulatum*. It has been shown to respond to a wide range of environmental stresses, including exposure to DNA damaging chemicals, heat-shock, osmotic stress, oxidative stress, DNA replication stress and exposure to antifungals (McClanahan and McEntee, 1986; Treger and McEntee, 1990; Miralles and Serrano, 1995; Karababa et al., 2004; Hromatka et al., 2005; Setiadi et al., 2006; Hao et al., 2009; Tkach et al., 2012; Mostafa and Awad, 2015). A recent study found that in *H. capsulatum* DDR48 confers some resistance to the antifungal azoles *via* the regulation of sterol biosynthesis (Reyes, 2019; Blancett et al., 2020), which is consistent with an increase in *ddr48* expression in azole-resistant isolates of *C. albicans* (Mostafa and Awad, 2015). DDR48 is also known to be required for *H. capsulatum* pathogenicity and survival in macrophages, likely due a role regulating the enzymes needed to detoxify reactive oxygen species (ROS) (Blancett, 2019; Blancett et al., 2020). Regulating complex pathways in response to environmental factors is perfectly consistent with a potential role in the regulation of secondary metabolites such as pleuromutulin, which are known to be produced under specific environmental conditions.

Silencing of *cprp via* the expression of an antisense construct generated a population of *C. passeckerianus* transformants exhibiting a pronounced reduction in pleuromutilin production, demonstrating that Cprp does indeed impact pleuromutilin biosynthesis. The variation in phenotypes observed is typical for antisense silencing, as the extent of silencing is impacted by many factors including the integration site and copy number of the silencing cassette. The transformation technique used in this work is known to result in multiple random integration events in *C. passeckerianus* (Kilaru et al., 2009), which may account for the observed variation in pleuromutilin titres. HPLC analysis confirmed a reduction in yield that was consistent with the bioassay results, providing further confidence that the observed impact on pleuromutilin production is genuine.

Attempts to complement an *S. cerevisiae ddr48* mutant (strain Y16748), as a means of investigating functional homology, were hampered by the lack of a clear mutant phenotype. No difference could be detected between the mutant and wild-type in terms of heat-shock response, and no spontaneous canavanine resistance was observed, removing it as a method of measuring spontaneous mutation rates. Since the initial report that DDR48 is involved in spontaneous mutation rates (Treger and McEntee, 1990), a subsequent study failed to detect any such phenotype despite testing multiple loci and conducting sequencing analysis (Roche et al., 1995), so this potential phenotype was not pursued further. The previous reports of increased sensitivity to osmotic stress (Yoshikawa et al., 2009) could also not be replicated in this study.

One unexpected finding from this work was the impact of DDR48 and Cprp on growth rates. The *S. cerevisiae ddr48* mutant appeared to have a slightly increased growth rate, and although this difference was small, it is consistent with previous findings. Breslow et al. (2008) developed a highly sensitive method for testing competitive fitness in *S. cerevisiae* and of 4,204 deletion strains tested, 45% had observable growth defects but only a few strains, including a *ddr48* mutant, exhibited a slight growth advantage. Breslow and colleagues observed that many of the strains found to have an increased growth rate contained mutations in genes involved in nutrient-signaling pathways, suggesting that they may help to regulate or limit growth when resources are limited to minimize resource depletion (Breslow et al., 2008). Consistent with the increase in growth rate upon deletion of *ddr48* in *S. cerevisiae* is an increase in colony size seen for many *cprp*-AS transformants, which had an average colony diameter of 112.4% of wild-type, with one strain recording a colony diameter that was 129% of wild-type. Conversely, when either DDR48 or Cprp were highly expressed in *S. cerevisiae* strain Y16748, a dramatic decrease in growth rate of ~60% was observed. If DDR48 is involved in nutrient signaling, overexpression may be expected to cause the yeast cells to enter the stationary phase, which is characterized by cell cycle arrest and normally occurs when available nutrients have been exhausted (Werner-Washburne et al., 1993).

Although no studies have specifically investigated a role for DDR48 homologs in nutrient signaling, there is significant data which supports this supposition. *Ddr48* expression is impacted by a number of regulators involved in nutrient signaling including AFT1 (iron deprivation); CST6 (non-optimal carbon source utilization) and MIG1 (glucose repression) (Hu et al., 2007). Searching the Expression Atlas database (Papatheodorou et al., 2018) reveals that *ddr48* is also downregulated in *ura2* and *gln3* deletion mutants; Ure2 and Gln3 both being key regulators in nitrogen catabolite repression. *Ddr48* has also been shown to be upregulated in a *S. cerevisiae gis1* mutant strain (Zhang et al., 2009); Gis1 being a transcription factor which is thought to be involved in shift from exponential growth to the stationary phase in response to nutrient availability. In line with this, Kusch et al. (2008) identified DDR48 as being one of the 50 most abundant proteins during stationary growth in *C. albicans*. In filamentous fungi, the link between secondary metabolism and the stationary phase has been so well-established that the stationary phase has been described as “the secondary metabolite producing growth phase” (Keller, 2019). The identification of *cprp* as being upregulated during pleuromutilin production may suggest that under production conditions, cultures of *C. passeckerianus* enter into the stationary phase, although this has not been explicitly tested. Cprp playing a role in coordinating entry into the stationary phase with the regulation of processes such as secondary metabolism is one plausible explanation for the impact of *cprp* downregulation on both growth rate and pleuromutilin biosynthesis.

In different fungal species, DDR48 has been reported as both a cell wall protein (Tonouchi et al., 1994) and as being localized to the cytoplasm (Huh et al., 2003). To investigate the localization of Cprp, a Cprp-GFP fusion protein, consisting of the full Cprp peptide fused to GFP, was successfully expressed in *C. passeckerianus*. Confocal microscopy provided clear evidence of localization adjacent to the septa with some increased fluorescence observed at the periphery of the cell. The localization appears to be asymmetrical, differing on either side of the septa, with a pattern which is intriguingly reminiscent of subcellular compartments formed due to cytoplasmic streaming in filamentous fungi (Pieuchot et al., 2015) (Supplementary Figure 8). There is precedent for fungal proteins involved in stress responses localizing to the septa in filamentous fungi. AoSO, a protein from *Aspergillus oryzae* was found to be dispersed throughout the cytoplasm under normal growth conditions but quickly localized to the septa following various stresses, including extreme temperatures, very acidic or alkaline pH, nitrogen and carbon depletion and physical depletion (Maruyama et al., 2010). AoSO is now known to be a component of stress granules in *A. oryzae* (Huang et al., 2013), which are non–membrane-bound assemblies of protein and RNA (known as messenger ribonucleoproteins; mRNPs) that form in response to a variety of environmental stresses and which regulate gene expression.

Finally, we have established that DDR48 and Cprp belong to a family of similar repetitive proteins encoded by much of the higher fungi. These proteins all contain a repetitive motif with a putative RNA binding sequence (S-Y-G) and are predicted to be intrinsically disordered proteins (IDPs) which contain prion-like domains (PrLDs). IDPs play various cellular roles including in transcriptional regulation, where their specific function is aided by their conformational flexibility. Not only are IDPs known to play an important role in interactions with nucleic acids, but intrinsically disordered sequences have recently been reported as important drivers of liquid-liquid phase separation (LLPS), a feature required for the formation of RNA granules such as stress granules. Aromatic residues such as tyrosine, which is at the core of the repetitive motif of all Cprp-like proteins, are thought to be particularly important for LLPS (Lin et al., 2017). The S-Y-G motif provides another tentative link to RNA granules, as a similar motif has been characterized in a family of RNA-binding proteins from humans which are known to be components of RNA granules and which are implicated in a spectrum of diseases (Mitsuhashi et al., 2017). A potential role for DDR48 proteins in RNA-mediated regulation is consistent with a study which found that DDR48 overexpression in *S. cerevisiae* resulted in a 63% decrease in RNA accumulation (Li et al., 2008). Additionally, Cprp and DDR48 both contain a PTZ00110 helicase domain, a domain seen in DEAD-box ATP-dependent RNA helicases. Members of this enzyme family are known to participate in every aspect of RNA metabolism (Cordin et al., 2006), and are known to localize to RNA granules (Hilliker, 2012). Although entirely speculative at this point, notional ATP-dependent RNA helicase activity is consistent with the previous finding that DDR48 from *S. cerevisiae* exhibits ATP hydrolysis *in vitro* (Sheng and Schuster, 1993).

## Conclusion

Cprp is an unusual repetitive protein which is abundantly expressed under pleuromutilin production conditions, contains multiple copies of a (N/D)(N/D)(N/D)SYGS motif, and which shares significant sequence similarity with the stress-response protein DDR48 from yeast. Silencing of *cprp* confirmed that this protein impacts pleuromutilin biosynthesis, and localization studies showed clear accumulation of Cprp adjacent to the septa. Although complementation of a *ddr48* mutant was hampered by the inability to reproduce previously reported mutant phenotypes, DDR48 and Cprp caused a clear and identical overexpression phenotype, namely a reduction in growth rate of ~60%. This phenotype is compatible with previous evidence for a role in nutrient signaling, a role which could logically align with a protein having an impact on both growth rates and secondary metabolite (SM) biosynthesis—SM biosynthesis being known to be frequently regulated by nutrient availability, and in particular entry into the stationary growth phase. A bioinformatic analysis of DDR48 and Cprp predicted that they are both intrinsically disordered proteins, consisting mostly of prion-like domains (PrLDs), two features commonly seen in RNA binding proteins, and particularly in proteins involved in the formation of RNA granules. They also share a core [G/S]-Y-[G/S] motif with a family of RNA-binding proteins from humans. As a family of fungal proteins which appear to regulate both primary and secondary metabolism in response to a wide range of environmental factors, DDR48 and Cprp-like proteins undoubtably warrant further investigation.

## Materials and Methods

### Fungal Strains and Growth Conditions

*Clitopilus passeckerianus* strain DSMZ1602 was used throughout these experiments. This strain was maintained at 25°C on Potato Dextrose Agar (PDA 24 g/L potato dextrose, 15 g/L agar). Potato Dextrose Broth (PDB: 24 g/L potato dextrose) was used for making *C. passeckerianus* glycerol stocks.

*Saccharomyces cerevisiae* strain BY4742 (*MAT*α, *his3*Δ*-1, leu2*Δ*-0, lys2*Δ*-0, ura3*Δ*-0*) (Brachmann et al., 1998) was used for all yeast recombinations and as the “wild-type” strain in complementation studies, along with a mutant *S. cerevisiae* strain (*BY4742, YMR173w::kanMX4*) which was obtained from EUROSCARF (Accession number: Y16748). *Bacillus subtilis* strain ATCC 6633 was used for all plate-based bioassays for antibiotic activity. For cloning, chemically competent cells were prepared using DH5α^TM^ *E. coli* cells.

### Media

#### C. passeckerianus

All cultures of *C. passeckerianus* were incubated at 25°C, with liquid cultures grown with shaking at 230 rpm. **CSO1-A** (4 ml/L rape seed oil, 50 g/L glucose, 12.5 g/L yeast extract, 1.0 g/L KH_2_PO_4_, 0.5 g/L MgSO_4_.7H_2_O, 0.7 g/L Ca(N0_3_)_2_.4H_2_O, 0.1 g/L NaCl, 0.05 g/L FeSO_4_.7H_2_O; pH 6.2). *C. passeckerianus* was grown in CSO1-A prior to transformation. **PDAS** (24 g/L potato dextrose broth, 15 g/L agar, 205 g/L sucrose; pH 6.3) was used as regeneration medium for *C. passeckerianus* protoplasts after transformations. **PVS** (8 g/L rape seed oil, 35 g/L spray dried corn liquor, 15 g/L glucose, 5 g/L calcium carbonate) was used as the seed medium for HPLC analysis of *C. passeckerianus*. **CGC** (50 g/L glucose, 5 g/L spray dried corn steep liquor, 2 g/L calcium carbonate) was used as the production media for HPLC analysis of pleuromutilin production. **Tryptic Soy Agar-b** (**TSAb**: 30 g/L tryptic soy broth, 20 g/L agar) was used as the base agar on which to grow *C. passeckerianus* for pleuromutilin bioassays.

#### S. cerevisiae

*S. cerevisiae* was grown at 28°C either on agar plates or in liquid media shaking at 220 rpm.

**YPDA** (10 g/L yeast extract, 20 g/L peptone, 20 g/L D-glucose, 20 g/L agar) was used for growth and maintenance of *S. cerevisiae*. **YPD** (as above but without agar) was used for growth rate assays for strains Y10000 and Y16748. **Yeast Synthetic Drop-Out Medium** (**YSDOM**: 5 g/L casein hydrolysate, 6.7 g/L yeast nitrogen base w/o amino acids, 20 mg/L adenine, 20 mg/L tryptophan, 20 g/L agar, 100 ml of sterile 20% glucose (w/v) added after autoclaving) was the selective medium for *S. cerevisiae* post transformation. **Yeast Synthetic Drop-Out Medium-Gal** (**YSDOM-G**: 5 g/L casein hydrolysate, 6.7 g/L yeast nitrogen base w/o amino acids, 20 mg/L adenine, 20 mg/L tryptophan, 20 g/L agar, 100 ml of sterile 20% galactose (w/v) added after autoclaving) was used for the analysis of *S. cerevisiae* strains overexpressing either DDR48 or Cprp, as galactose induces expression of the pGAL1 promoter.

#### E. coli

All *E. coli* strains used were grown at 37°C on either solid agar plates or in liquid media with shaking at 225 rpm. *B. subtilis* was incubated at 30°C either as an overlay (static) or with shaking at 200 rpm. **Luria-Bertani Broth** (**LB**: 10 g/L NaCl, 10 g/L tryptone, 5 g/L yeast extract, pH 7) and **Luria-Bertani Agar** (**LBA**: LB supplemented with 15 g/L agar) were used for standard propagation. **Tryptic Soy Broth** (**TSB**: 30 g/L tryptic soy broth) was used to grow a *B. subtilis* culture for the preparation of *B. subtilis* spores used in bioassays. **Tryptic Soy Agar-g (TSAg**: 30 g/L tryptic soy broth, 5 g/L agar, 10 g/L glucose) was supplemented with 1 ml/L of *Bacillus subtilis* spore suspension (~4.5 × 10^9^ spores/ml) and 7.5 ml/L of 4% TTC (2,3,5-Triphenyl-2H-tetrazolium chloride) and used as the overlay for bioassays.

#### Media Supplements

Hygromycin B (Duchefa) was added to either PDAS (regeneration medium−600 μg/ml overlay) or PDA (maintenance medium−50 μg/ml) to select for fungal transformants. Ampicillin (Melford) was used to select for *E. coli* transformants at a working concentration of 50 μg/ml.

### PCR

Amplification of fragments for plasmid construction were carried out using the proof-reading polymerase KAPA HiFi HotStart (Roche). Analytical PCRs were performed using Biomix Red (Bioline). Primers used in this study are listed in Table 2.

**Table 2:** Primers used in this study.

cprp-antisense-F	*TTTGATGATTTCAGTAACGTTAAGTGGATC*GAACAACTAGCTCTTGCATCTC
cprp*-*antisense-R	*CTCCCATCTACACACAACAAGCTTATCGCC*AGTTCGAATACGAATTCTTGA
Agaricus-Gpdprom-F	GAAGAAGAATTCAGAGGTCCG
cprpAS-int-F	CACCTCCTCGAACCAGAGTGA
CP1-tub-F	GTCAACATGGGTTAGTGGCC
CP1-tub-R	GCAGCGACCTAACCAAACAC
*Hind*III_DDR48F	AAGCTTAGTGAACGAAAAAAACAGCAATGG
*Xba*I_DDR48R	TCTAGATATCGAAGACATCCAAAAACTTAG
*Hind*III_DDR48-cprpF	AAGCTTagtgaacgaaaaaaacagcaATGTCTTACAACCGCGACAAC
*XbaI*_cprpR	TCTAGATTAGTTCGAATACGAATTCTTG
cprp-fusion-F	*ACGGATTAGAAGCCGCCGAGCGGGTGACAG*TTGTGGCAAGGTGGCAGATC
cprp-fusion-R	*GGTGAACAGCTCCTCGCCCTTGCTCACCAT*GTTTCCGACTGAAAGATGAG
GFP-cprpfusion-F	*TTAATAACCTCTCATCTTTCAGTCGGAAAC*ATGGTGAGCAAGGGCGAGGA
GFP-cprpfusion-R	*TTGATGATTTCAGTAACGTTAAGTGGATCC*TTACTTGTACAGCTCGTCCA
cprp-Probe-F	AACACCTCCTCGAACCAGAGCG
cprp-Probe-R	AGTTCGAATACGAATTCTTGA

*Compound primers with tails (in italics) were designed to allow for yeast recombination. Introduced restriction sites are underlined. The sequence in lowercase in primer HindIII_DDR48-cprpF is to introduce 20 bp of the DDR48 5′UTR to the amplified cprp coding sequence*.

### Restriction Enzyme Digests

Restriction enzyme digests were used to linearise plasmids prior to yeast recombination and to analyse plasmids after construction. All enzymes and buffers were purchased from Thermo Scientific and used according to the manufacturers' instructions.

### Ligations

Ligations were conducted using Quick-stick ligase from Bioline and set up as follows: 4 x Quick-Stick Buffer (2.5 μl), T4 DNA ligase (0.5 μl), linearised vector backbone (X μl), insert (X μl) and water to 10 μl, with the backbone and insert added at a molar ratio of 3:1 with a maximum total weight of 100 ng. The ligation reactions were incubated at room temperature for 20 minutes before transforming the entire 10 μl into competent *E. coli* (DH5-α).

### Fungal DNA Preparation

DNA extractions for the purpose of fragment amplification for building constructs and diagnostic PCR analysis were carried out as described by Liu et al. (2000).

### Fungal RNA Preparation

All RNA preparations were done using the RNeasy Mini Kit (Qiagen) according to the manufacturer's protocol. For northern blot analysis, total RNA was extracted from 5-day old *C. passeckerianus* mycelia grown in PDB using the RNeasy Mini Kit (Qiagen) according to the manufacturer's protocol. Gel electrophoresis and spectrophotometric quantification were used to check quality and to calculate RNA concentrations.

## Bioinformatics

Analysis of sequence chromatograms was conducted using Sequencher 4.10.1. Protein alignments were conducted using Clustal Omega (Sievers and Higgins, 2018). Blast2Go (Götz et al., 2008) was used to conduct large-scale blast searches using the blastx algorithm and automatically assign Gene Ontology (GO) terms to the EST sequences. To identify putative Cprp homologues the blastp algorithm (Altschul et al., 1990) was used to interrogate the NCBI nr database. MEGA-X (Kumar et al., 2018) was used to produce a phylogenetic tree of Cprp/DDR48-like proteins using the neighbor-joining method, with bootstrap test percentages of 1,000 replicates shown next to the branches. To produce sequence logos, the online tool WebLogo was used (Crooks et al., 2004).

### Antibiotic Bioassays and HPLC Analysis

#### Plate-Based Bioassays

Fungal strains were point inoculated onto the center of a petri dish containing 25 ml of tryptic soy agar (TSA) and allowed to grow for 5 days at 25°C. Bioassays were then conducted as previously described (Hartley et al., 2009).

#### HPLC Analysis

*C. passeckerianus* was grown in 50 ml CGC at 25°C with 230 rpm shaking for 5 days. Mycelia was then homogenized for 3 min using a polytron homogeniser. 3 ml of acetonitrile was added to 1 ml of homogenized culture and shaken for 15 min (1,000 rpm). 1 ml of sample was then centrifuged at 16,000 × g for 10 min to pellet particulate matter and the supernatant was collected for HPLC analysis.

Pleuromutilin titer was calculated using the HP1050 HPLC system fitted with a 260 × 4.6 mm C18 column and a C18 guard column. The mobile phase used was 55% acetonitrile with a flow rate of 1 ml/min and a column temperature of 30°C. The absorbance was measured at a wavelength of 205 nm and the retention time for pleuromutilin was ~6 min. During each run a pure pleuromutilin standard of 1 mg/ml was used as a control. Titres were quantified by using a dilution curve based on a range of pleuromutilin standards (Supplementary Figure 7).

#### PEG-Mediated Transformation of *C. passeckerianus* Protoplasts

*C. passeckerianus* protoplasts were prepared and transformations were carried out as described previously (Kilaru et al., 2009). 48 h after the transformation, transformants were selected by the addition of a 5 ml PDAS (PDA plus 0.6 M sucrose, pH 6.5) overlay containing 600 μg/ml hygromycin B (Duchefa) to the regeneration agar (PDAS) plates. Once transformants appeared (1–2 weeks), they were individually subcultured onto PDA plates containing 50 μg/ml hygromycin B.

#### *S. cerevisiae* Transformation

The *S. cerevisiae* transformation protocol was modified from Raymond et al. (1999). 50 ml of YPD was inoculated with a *S. cerevisiae* overnight starter culture and incubated at 30°C for 5 h with 230 rpm shaking. Cells were harvested by centrifugation (2,200 × g for 5 min), washed once with SDW and resuspended in 300 μl SDW. To 50 μl of these cells, 50 μl of denatured (heated to 95°C for 2 min) salmon sperm DNA (2 μg/μl), and approximately 1 μg of transforming DNA were added and mixed. 272 μL Lithium acetate / PEG premix [240 μl 50% PEG 4000 (w/v in H_2_O) and 32 μL 1 M Lithium acetate] was added, mixed gently and incubated at 30°C for 30 min. The cells were heat shocked at 45°C for 15 min, pelleted by centrifugation at 2,000 rpm for 2 min, and resuspended in 200 μl H_2_O before plating out on yeast synthetic drop out medium (YSDOM).

#### Yeast Recombination

Plasmids were constructed using yeast homologous recombination. To achieve this, *S. cerevisiae* was transformed with equal molar amounts of a linearised backbone and the fragments to be recombined (typically 20–200 ng). Fragments were amplified using compound primers containing tails with homology to the backbone or neighboring fragment, to allow for homologous recombination. The resulting plasmids were extracted from *S. cerevisiae* using the Zymoprep™ Yeast Plasmid Miniprep I kit, according to the manufacturer's instructions and 5 μl was transformed into *E. coli* for plasmid rescue and propagation (see below).

#### *E. coli* Transformations

The preparation of chemically competent DH5-α *E. coli* cells was based on that described by Nishimura et al. (1990). An overnight liquid culture DH5-α was used to inoculate 50 ml medium A (LB with 10 mM MgSO_4_.7H_2_O, 0.2% glucose) which was incubated in an orbital shaker at 37°C, 225 rpm, until the culture reached mid-logarithmic phase (~2.5 h). Following 10 min incubation on ice, the cells were harvested at 1,500 × g for 10 min at 4°C and resuspended in ice-cold medium A (0.5 ml). 2.5 ml of storage solution B (LB supplemented with 36% glycerol, 12% PEG 7,500 and 12 mM MgSO_4_.7H_2_O; filter sterilized) was added and mixed gently. Cells were divided into 100 μl aliquots and stored at −80°C.

For plasmid construction, 5 μl of yeast plasmid extractions was added to 100 μl of chemically competent DH5-α cells and was incubated on ice for 30 mins. The cells were heat shocked in a 42°C water bath for 45 s and then cooled on ice for 2 min. 1 ml of LB was added and the cells were incubated at 37°C with 230 rpm for 30 min before plating out on LBA with appropriate antibiotics.

#### Construction of the *cprp* Silencing Construct

Primers cprp-antisense-F and cprp-antisense-R were used to amplify the *cprp* antisense fragment from a cDNA clone (cp_cgc_19_E04). This produced a 1,033 bp product (plus 60 bp of compound tails introduced by the primers) which included the 843 bp coding sequence for *cprp*, a 42 bp section of the 5′ UTR and the entire 148 bp 3′ UTR. Plasmid pYES-hph-CBXgene (Bailey et al., 2016) was digested with *Xho*I and *Sac*I to remove the CBX gene, and the *cprp* antisense fragment was recombined into the CBX cassette in the antisense orientation using yeast homologous recombination. This placed the *cprp* antisense fragment under the control of the constitutive GpdII promoter. The resulting plasmid was named pYES-hph-*cprp*AS.

#### Construction of Complementation Plasmids

Constructs for the complementation of a *ddr48* mutant strain were all constructed using the yeast expression vector; pYES2-19.

#### pYES2-DDR48

The entire open reading frame of *ddr48* plus 20 bp of the 5′ UTR, was amplified from *S. cerevisiae* strain BY4742 using primers *Hind*III_DDR48F and *Xba*I_DDR48R. The resulting product was cloned in pJET1.2, sequenced, and confirmed as being identical to the *ddr48* sequence within the genome sequence for *S. cerevisiae* strain S288C, the strain from which BY4742 was derived. *Ddr48* was then excised from pJET1.2-DDR48 using the restriction enzymes *Hind*III and *Xba*I and ligated into pYES2-19, producing the plasmid pYES2-DDR48.

#### pYES2-CpcDNA and pYES2-CpgDNA

To construct pYES2-CpcDNA, the primers *Hind*III_DDR48-cprpF and *XbaI*_cprpR were used to amplify the coding sequence of *cprp* from a cDNA clone (cp_cgc_19_E04) of *C. passeckerianus* cDNA library. Primer *Hind*III_DDR48-cprpF contains 20 bp of the *ddr48* 5′ UTR from *S. cerevisiae*, to improve transcription in yeast. The resulting PCR product was cloned into pJET2.1, sequenced, digested out using *Hind*III and *Xba*I, and ligated into pYES2.

pYES2-CpcDNA was constructed exactly as above but rather than using a cDNA clone as the template for amplification of *cprp* coding sequence, the entire *cprp* gene from *C. passeckerianus* genomic DNA was amplified using primers *Hind*III_DDR48-cprpF and *XbaI*_cprpR.

#### Construction of the cprp-GFP Fusion Cassette

To identify the promoter region for Cprp, the genomic region upstream was annotated. A highly conserved gene was identified 1,050 bp upstream of the *cprp* start codon which encodes a 3-hydroxyanthranilate 3,4-dioxygenase. As 1 kb is typically accepted as a suitable length to contain a fungal promoter, and it is unlikely that the promoter region extends into the coding region of the adjacent gene, this region was taken to be sufficient to drive expression. A likely TATA box (TATAAAA) was identified 101 bp upstream of the *cprp* start codon.

**Figure 7:**
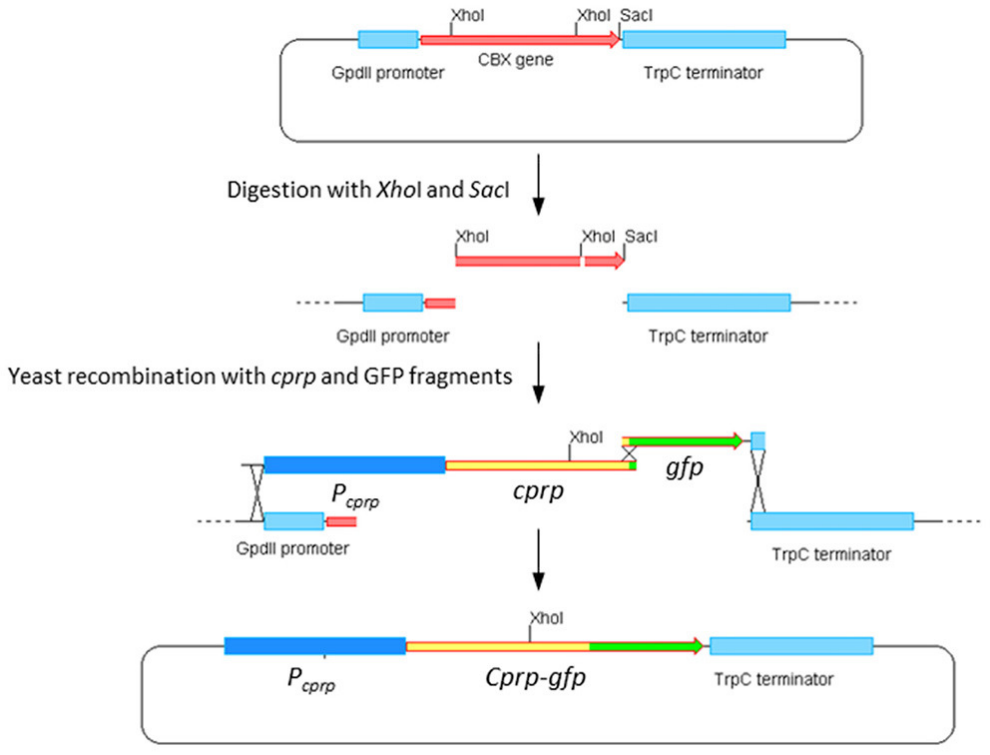
Construction of pYES-hph-cprpGFP.

Yeast homologous recombination was used to build the desired *cprp*-*GFP* fusion cassette and the strategy is summarized in Figure 7. Compound primers cprp-fusion-F and cprp-fusion-R were used to amplify the entire promoter region and *cprp* ORF. cprp-fusion-F was designed to bind to the 20 bp at the 3′ end of the adjacent gene to ensure the entire intergenic region was amplified and to avoid any polymorphisms within the promoter region. To allow for recombination, the 5′ end of this primer consisted of the 30 bp upstream of the *Gpd*II promoter in the plasmid pYES-hph-CBXgene so that during yeast recombination the promoter region of *cprp* would replace the *Gpd*II promoter. cprp-fusion-R consists of the last 20 bp of the *cprp* ORF, minus the stop codon and the first 30 bp of the ORF for GFP so that once recombined, the GFP start codon would replace the *cprp* stop codon giving an in-frame fusion. GFP was amplified from plasmid p004iGM3 (Burns et al., 2005) using the primers GFP-cprpfusion-F and GFP-cprpfusion-R. These are also compound primers. GFP-cprpfusion-F binds to the first 20 bp of the *gfp* ORF, adding the last 30 bp of *cprp* (minus the stop codon) to the 5′ end of the product. The reverse primer was designed to bind to the last 20 bp of the *gfp* ORF, including the stop codon, adding the first 30 bp of the *Trp*C terminator to the 3′ end of the amplified product. This was to allow *gfp* to recombine into pYES-hph-CBXgene with the *gfp* stop codon followed by the *Trp*C terminator.

#### Plasmid Purification and Sequencing

Qiagen plasmid preparation kits were used for all plasmid DNA purification. For sequencing and PCR, 5 ml of LB was inoculated with a single bacterial colony and incubated overnight followed by an extraction using the Qiagen Mini kit according to the manufacturer's instructions. For PEG-mediated transformations large quantites of very clean DNA were required and the Qiagen Midi Kit was used with 50 ml of overnight culture, again according to the manufacturer's instructions. Single-pass sequencing was used to confirm the sequences of novel constructs and was conducted by LCG genomics (previously known as AGOWA).

### Northern Blot Analysis

#### Membrane Preparation

4 μg of total RNA was denatured for each sample, separated on a 0.8% agarose gel and transferred onto Hounds-N nylon membrane (GS Healthcare) overnight by capillary action. RNA was fixed to the membrane by UV crosslinking using a UVP CL-1000 cross-linker at 1,200 kjoules.cm^−1^.

#### Hybridisation

The membrane was pre-wetted in SDW, followed by 5 × SSC then prehybridised in 50 ml Church buffer at 65°C for at least 4 h with slight shaking. After this time the buffer was discarded and replaced with 20 ml of fresh Church buffer. A *cprp* probe of 301 bp was generated by PCR using cprp-Probe-F and cprp-Probe-R and labeled with [α-^32^P]dCTP using the Ready-To-Go labeling kit (Amersham Pharmacia Biotech). The probes were then cleaned using illustra ProbeQuant^TM^ Micro Columns (GE Healthcare) according to the manufacturer's protocol. The labeled probe was denatured at 95°C for 2 min, cooled on ice for 2 min and added to the membrane. Hybridisation was carried out with slight agitation at 65°C for ~16 h.

#### Washing and Autoradiography

After overnight hybridization the membranes were washed at 65°C in a succession of buffers. This consisted of rinsing the membrane in 2 × SSC + 0.1% SDS then soaking in 2 × SSC + 0.1% SDS with agitation for 5 min. This was then repeated with 1 × SSC + 0.1% SDS. During washing the membrane was monitored with a Geiger counter until the rate was ~50–100 counts per second. If counts remained high using the above buffers, 0.5 × SSC + 0.1% SDS and 0.1 × SSC + 0.1% SDS would be used. Washed membranes were placed RNA side down on saran wrap and excess solution was blotted away using filter paper. The membrane was then wrapped in the saran film and placed in a radiography cassette and exposed to autoradiography film at −80°C overnight. The radiography cassette was allowed to warm to room temperature to minimize static. The autoradiograph film was then placed in developing fluid for 5 min, rinsed in water and placed in fixing fluid for 5 min. After a final wash in water, the film was air dried.

#### Stock Solutions

The following solutions were used in Northern analysis.

20 X SSC: 175.3 g/L NaCl, 88.2 g/L sodium citrate - pH 7. Diluted with DEPC treated SDW to make 0.5 x, 2 x, 5 x, and 10 x SSC.

Church buffer: 200 ml phosphate buffer (171 ml of 141.96 g/L Na_2_HPO_4_ and 79 ml of 119.98 g/L NaH_2_PO_4_ - pH 7.2), 700 ml 10 % SDS, 2 ml of 0.5 M EDTA (pH 8). Made up to final volume of 1 L.

### *S. cerevisiae* Phenotypic Screening

#### Strain Y10000 (‘WT') vs. Y16748 (Δddr48)

Mutation rates, as measured by the spontaneous resistance to canavanine, were studied exactly as previously described by Treger and McEntee (1990). Responses to osmotic stress were tested using both agar spotting assays and growth rate in liquid cultures. For agar spotting assays, cultures were incubated overnight in liquid YPD medium, and diluted to OD_640_ = 1 with fresh medium. Drop tests were performed by spotting 5 μl of serially diluted cell suspension onto YPD Agar ± 1 M NaCl. To measure growth rates in liquid cultures, strains were grown in YPD overnight to saturation. The optical density of these starter cultures was checked and normalized to ensure equal inoculum. To ensure that varied cell morphology did not impact estimations of cell numbers, a haemocytometer was also used to check the cell concentration which was found to be ~3.1 × 10^8^ for both strains. 50 μl of the starter culture was then used to inoculate 5 ml volumes of fresh YPD containing a variety of NaCl concentrations and the optical density of the cultures was recorded after 16 h. To account for any difference in growth rate between the wild-type and mutant strain, the optical densities were calculated as a percentage of YPD cultures with no additional NaCl.

#### DDR48 and Cprp Overexpression Strains

Each strain was grown to saturation overnight in YSDOM. The optical density of these starter cultures was checked and normalized to ensure equal inoculum. To ensure that varied cell morphology did not impact estimations of cell numbers, a haemocytometer was also used to check the cell concentration which was found to be ~3.1 × 10^8^. 50 μl of the starter culture was used to inoculate 5 ml of fresh YSDOM-G (a medium containing galactose to induce expression from the pGAL1 promoter) with either 0 M or 0.5 M NaCl concentrations. After ~16 h the optical density of each culture was recorded.

#### Microscopy

Haemocytometry and GFP detection were carried out using a Leica DMLB microscope, with GFP screening carried out with excitation filters at 450–490 nm, dichroic filter at 510 nm and emission filter at 515 nm.

Confocal microscopy was carried out using the Leica SP5-AOBS confocal laser scanning microscope equipped with a 100 mW Ar laser, attached to a Leica DM I6000 inverted epifluorescence microscope. The 488 Argon (blue) laser line and I3 filter set were used for detection of GFP.

## Data Availability Statement

The datasets presented in this study can be found in online repositories. The names of the repository/repositories and accession number(s) can be found in the article/Supplementary Material.

## Author Contributions

AB and GF contributed to the design of the research and had supervisory roles. KdM-S performed the experimental work, interpreted data, and drafted the manuscript. All authors contributed to the article and approved the submitted version.

## Conflict of Interest

The authors declare that the research was conducted in the absence of any commercial or financial relationships that could be construed as a potential conflict of interest.
