# Supplementary material for: Cprp—An Unusual, Repetitive Protein Which Impacts Pleuromutilin Biosynthesis in the Basidiomycete Clitopilus passeckerianus
Source: Front Fungal Biol. 2021 Apr 6;2:655323. doi: 10.3389/ffunb.2021.655323 (PMC10512284; doi:10.3389/ffunb.2021.655323)
Supplement: Supplementary file 1 [file Data_Sheet_1.PDF]

Supplementary Material

Cprp - An unusual, repetitive protein which impacts pleuromutilin biosynthesis in the basidiomycete *Clitopilus passeckerianus*

Kate M. J. de Mattos-Shipley[1]*, Gary D. Foster[1] & Andy M. Bailey[1]*
[1] School of Biological Sciences, Bristol Life Sciences Building, University of Bristol, 24 Tyndall Ave, Bristol, BS8 1TQ, UK
* Correspondence: Kate.deMattos-Shipley@bristol.ac.uk, Andy.Bailey@bristol.ac.uk

Table of Contents

1.1 Supplementary Figures 2
1.2 Supplementary Tables 8
1.3 Supplementary Data 10
1.4 References 12

Table of Figures

Supplementary Figure 1: The impact of nitrogen source on pleuromutilin production 2
Supplementary Figure 2: An alignment of DDR48 and Cprp 2
Supplementary Figure 3: A PLAAC analysis of Cprp 3
Supplementary Figure 4: A PLAAC analysis of DDR48. 3
Supplementary Figure 5: Full bioassay data for cprp-AS transformants and controls. 4
Supplementary Figure 6: Northern blot analysis of *cprp*-AS (antisense) transformants 4
Supplementary Figure 7: Standard curves for pleuromutilin quantification 5
Supplementary Figure 8: Cytoplasmic streaming in filamentous fungi and localisation of Cprp 5
Supplementary Figure 9: Identification of putative DDR48 homologues from different fungal taxa 6
Supplementary Figure 10: PCRs to confirm integration of the *cprp*-AS cassette 7

Table of Tables

Supplementary Table 1: Two-tailed t tests corresponding to Figure 3D 8
Supplementary Table 2: Two-tailed t tests corresponding to Figure 4B 8
Supplementary Table 3: Two-tailed t tests corresponding to Figure 5B 8
Supplementary Table 4: An analysis of putative DDR48 homologues 9

frontiers

1.1 Supplementary Figures

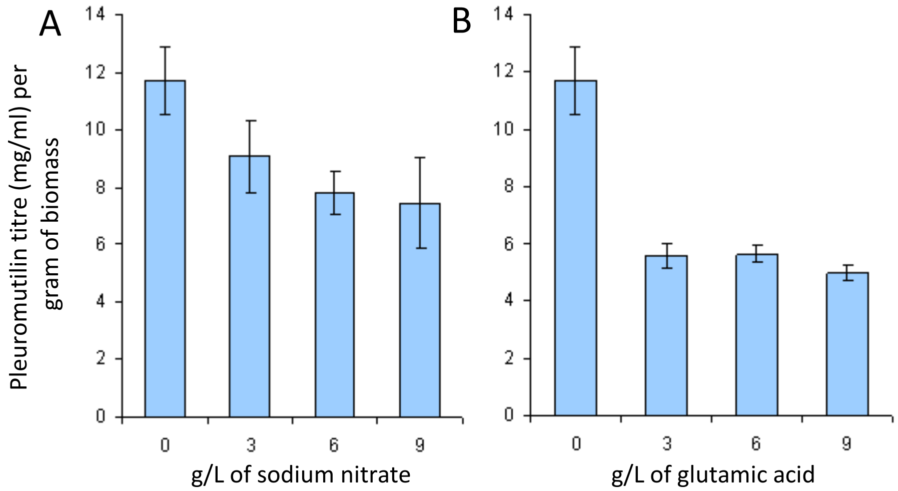

Supplementary Figure 1: The impact of nitrogen source on pleuromutilin production. Pleuromutilin yields were quantified in CGC cultures supplemented with either sodium nitrate (A) or glutamic acid (B). Triplicate cultures were analysed and standard error bars are shown.

```
sp|P18899.4|      MGLFDKVKQFANSNNNNNDSGNNNQGDYVTKAENMIGEDRVNQFKSKIGEDRFDKMESKV     60
Cprp              ------------------------------------------------------------     0


sp|P18899.4|      RQQFSNTSINDNDSNNNDSYGSNNNDSYGSNNNDS------YGSNNNDSYGS-----NNN     109
Cprp              --------MSYNRDNDNDSYGSGNNDSYGSRKNNDNNNTGGFNDNDNDSYGSKNKRDNNN     52
                          :. * .*:******.*******.:*:.      :..*:******     ***

sp|P18899.4|      DSYGSNNDDSYGSSNKKKSSYGSNNDDSYGSSNN--NDSYGS---NNNDSYGSNNNDSYG     164
Cprp              DSYGSSNNDSYGSSNRRKDDDN-DNSNSYGSNNNDNDNSYGSKTNKSNDSYGSSNNDSYG     111
                  *****.*:*******::*.. . :*.:****.**  ::****   :.******.******

sp|P18899.4|      SNNDDSYGSSNKNKSSYGSNNDDSYGSNNDDSYGSSNKKKSSYGSSNNDSYGSNNDDSYG     224
Cprp              SSNNDSYGSSNRRD------------NDNDNSYGSKNKDNDSYGSSNNDSYGSKKSDNYG     159
                  *.*:*******:..            .:**:****.**.:.************::.*.**

sp|P18899.4|      SNNNDSYGSNND----DSYGSSNKKKSSYGSNNDDSYGSSNNNDSYGSNNDDSYGSSNKN     280
Cprp              SSNNDTYGSSNRNDNNDSYGSSNKNKDS--------------------------------     187
                  *.***:***.*     ********:*.*

sp|P18899.4|      KSSYGSSSNDDSYGSSNNDDSYGSSNKKKSSYGSNNDDSYGSNNDDSYGSSNKKKSSYGS     340
Cprp              -----------------------------------NDSSYSSSNNDSYGSSNN------S     206
                                                     **.**.*.*:*******:      *

sp|P18899.4|      SNNDSYGSNNDDSYGSSNKKKSSYGSNNDDSYGSSNNNDSYGSNNDDSYGSSNRNKNSYG     400
Cprp              SNNDSYGSSNTSSYGSGNNNNNNNNT--------SSNQSDW--------V---DKGVSYA     247
                  ********.* .****.*:::.. .:        *.*:..:             :  **.

sp|P18899.4|      SSNYGSS---NNDDSYGSSNRGGRNQYGGDDDY  430
Cprp              SKQAGYNLDEATADKIGDGLREGFKKFGGGFGN  280
                  *.: * .    . *. *.. * * :::**. .
```

Supplementary Figure 2: An alignment of DDR48 from *Saccharomyces cerevisiae* (Accession number: P18899.4) and Cprp from *Clitopilus passeckerianus*. Alignment done using ClustalOmega (Sievers & Higgins, 2018). The two proteins demonstrate 59.94 % protein identity.

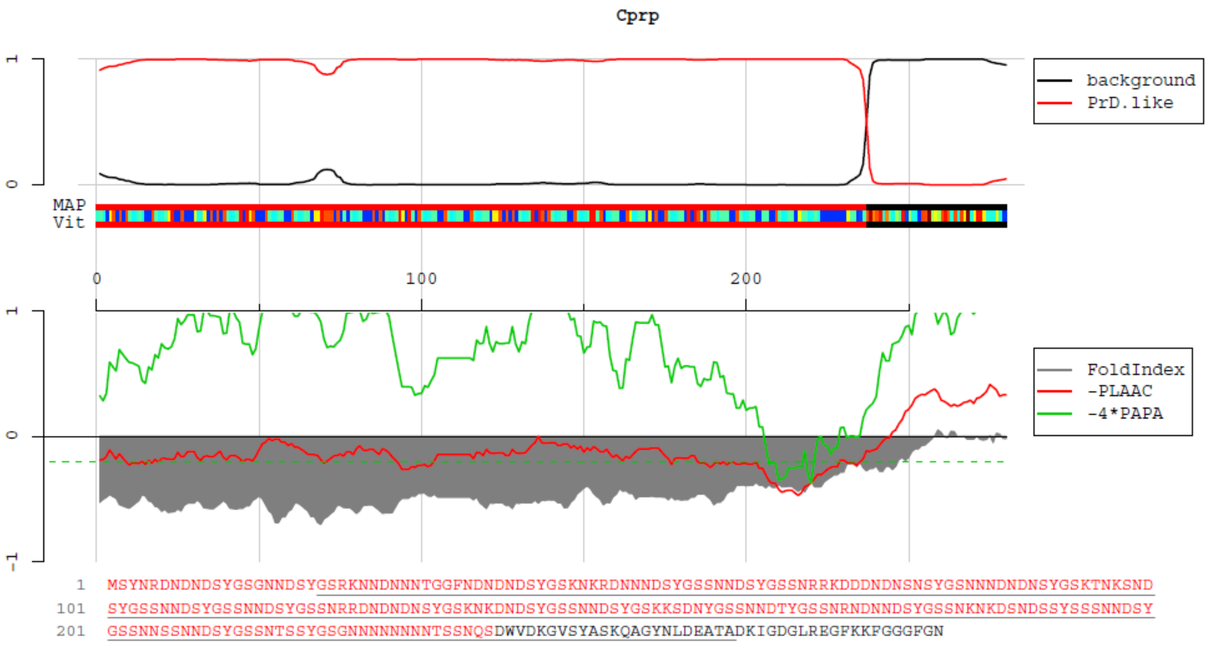

Supplementary Figure 3: A PLAAC analysis (Lancaster et al., 2014), showing that residues 1 – 237 of Cprp are predicted to form a prion-like domain.

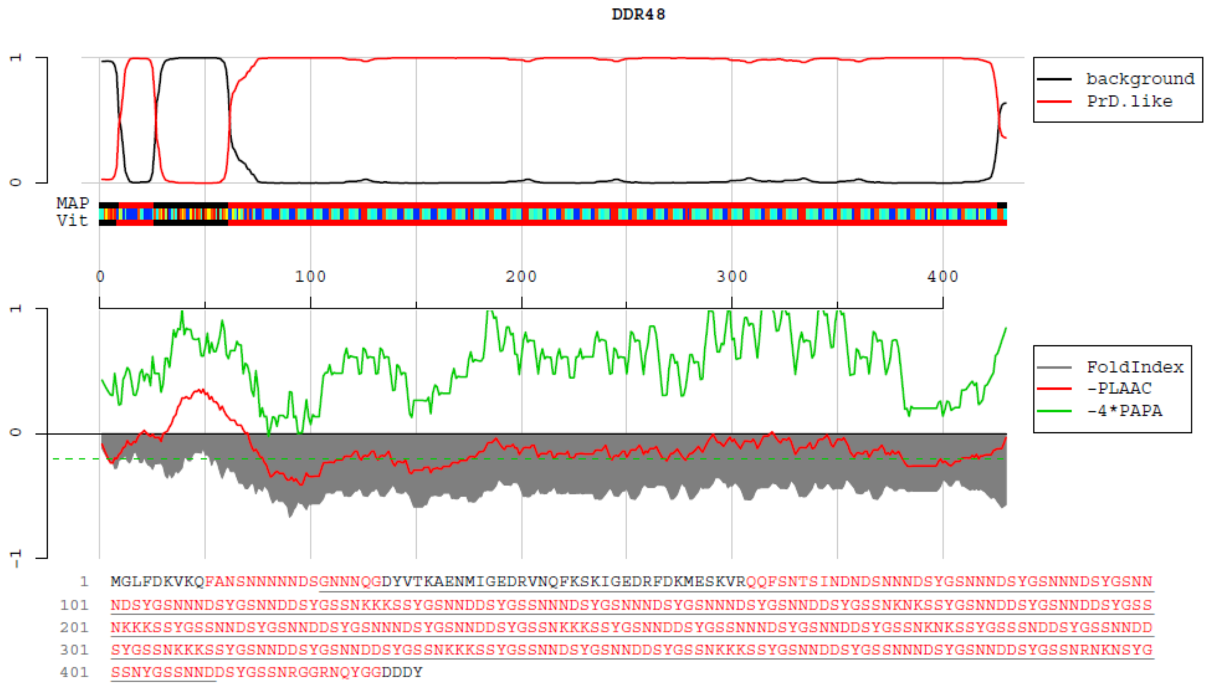

Supplementary Figure 4: A PLAAC analysis (Lancaster et al., 2014), showing that residues 10 – 26 and 62 - 426 of DDR48 from *S. cerevisiae* are predicted to form a prion-like domain.

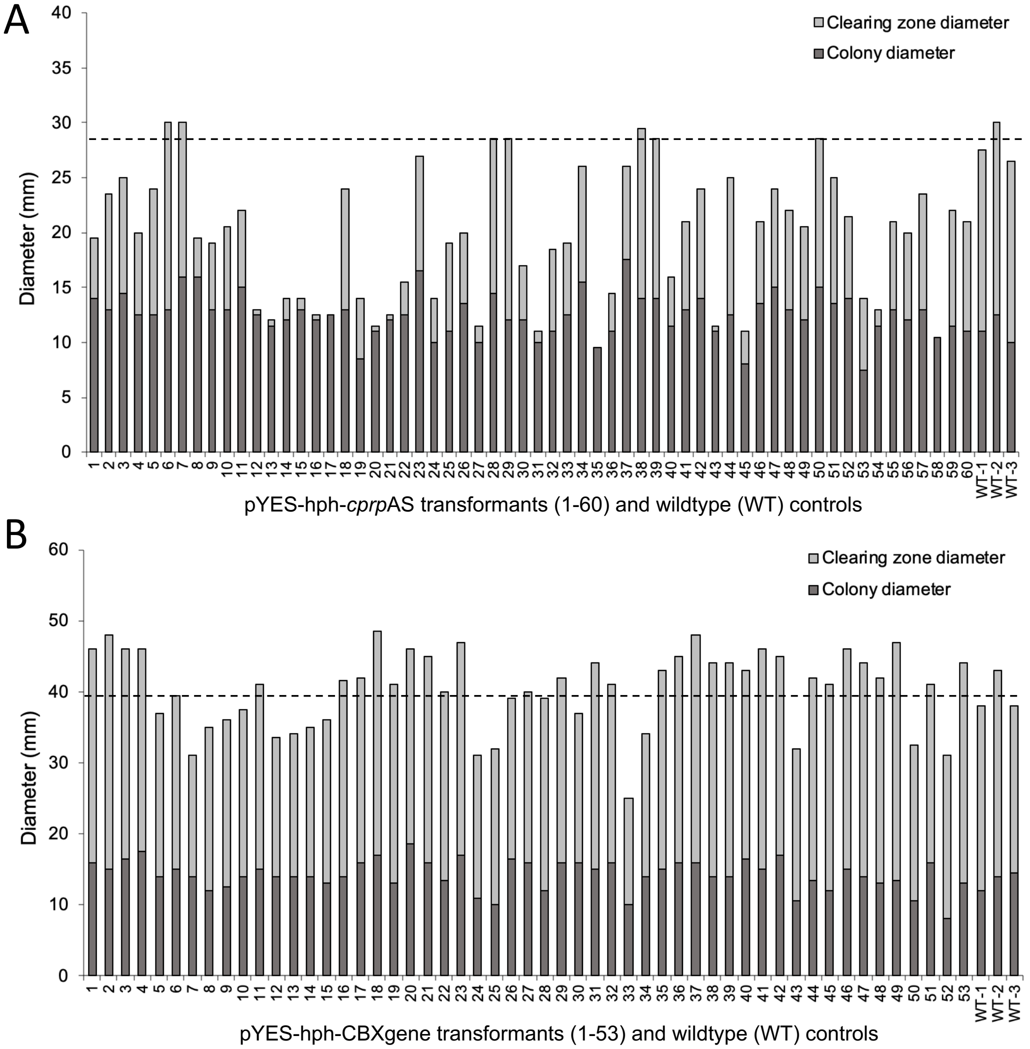

Supplmentary Figure 5: Full bioassay data for *cprp*-AS transformants (A) and a control set of transformants, lacking the cprp antisense silencing construct (B). The average clearing zone diameter for the wild-type triplicate assays is represented with a horizontal dashed line.

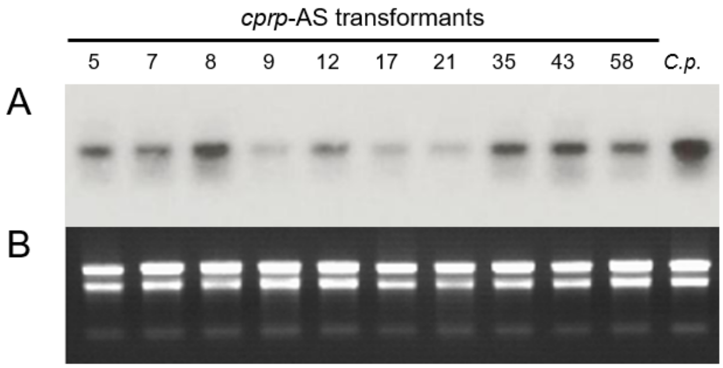

Supplementary Figure 6: Northern blot analysis confirmed silencing in a selection of *cprp*-AS (antisense) transformants. **A**) Autoradiograph showing a reduced intensity signal for silenced lines compared to the wild type. **B**) Ethidium bromide visualization of total RNA showing equal loading.

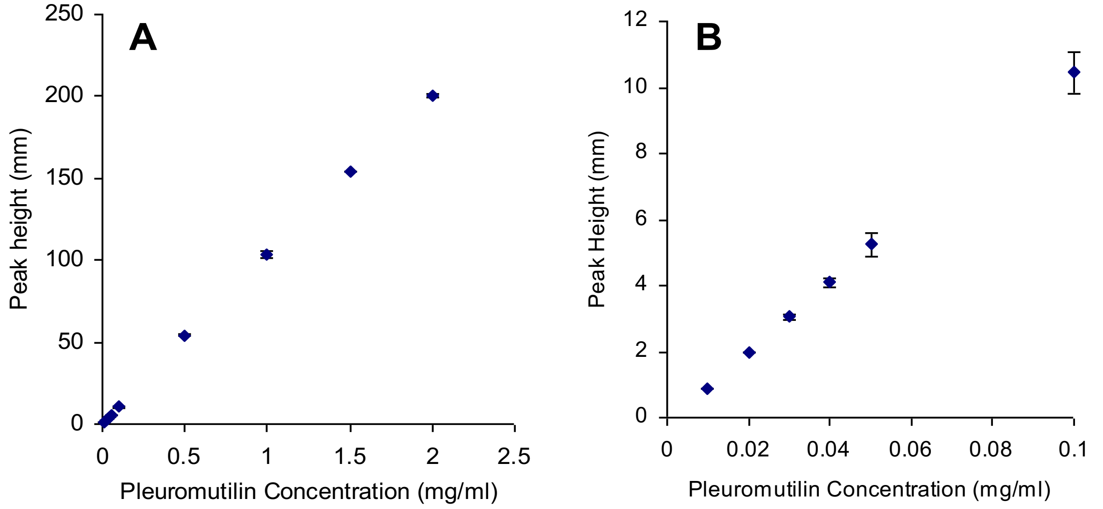

Supplementary Figure 7: Standard curves showing peak height on HPLC chromatograms for pleuromutilin standards of varying concentrations showing a clear linear relationship. The relationship is linear up to a concentration of 2 mg/ml (**A**) and pleuromutilin is detectable down to 0.01 mg/ml (**B**).

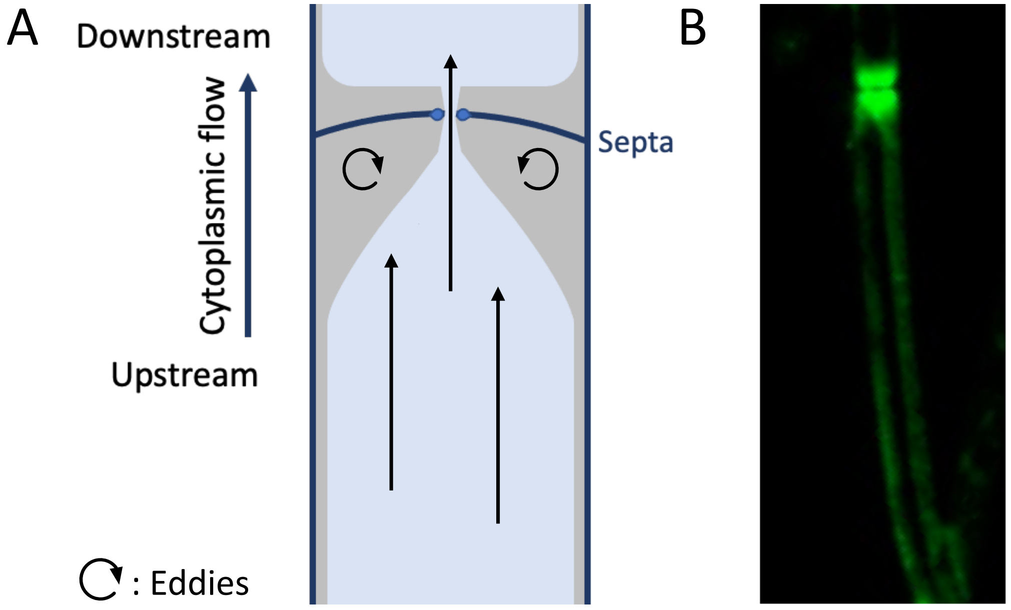

Supplementary Figure 8: A) Cytoplasmic streaming in the hyphae of filamentous fungi has been shown to create microfluidic eddies that act as subcellular compartments (in grey). Adapted from Pieuchot et al. (2015). B) Localisation of a Cprp-GFP fusion protein in hyphae of *Clitopilus passeckerianus* appears to be asymmetric on either side of the septa in a pattern that is reminiscent of cytoplasmic streaming.

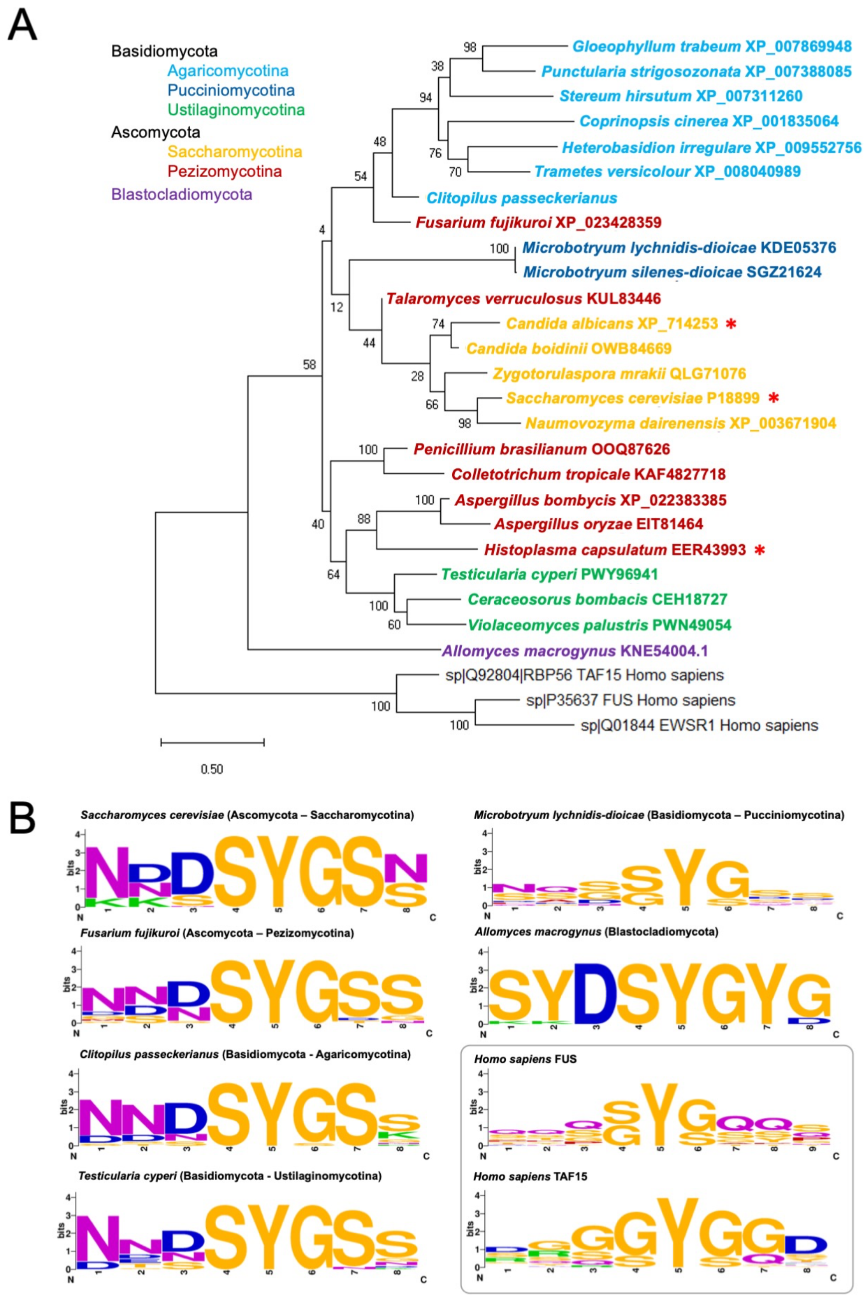

Supplementary Figure 9: A) A tree of Cprp/DDR48-like proteins identified from a range of fungal species, generated using Mega-X (Kumar et al., 2018). The known DDR48 proteins from *S. cerevisiae*, *C. albicans* and *H. capsulatum* are marked with '*'. Proteins from *Homo sapiens* containing the [G/S]Y[G/S] motif were also included in this analysis and act as an outgroup. **B**) Sequence logos for a selection of proteins, showing the conserved [G/S]Y[G/S] motif and more or less varied neighbouring residues. The number of motifs present in individual proteins varied and can be seen in Supplementary Table 4.

Note 1: The *Fusarium fujikuroi* protein sequence used in this analysis and listed Supplementary Table 4 (XP_023428359.1) is from an annotation conducted by Wiemann et al. (2013). It is considered to be homologous to Gsr1 (Teichert et al., 2004), as although the annotation (and therefore protein sequences) differ, the nucleotide sequences have 100 % identity. As the Wiemann et al. annotation is more recent and based on a study including transcriptomic data, it is considered to be more reliable than the original annotation of Gsr1.
Note 2: The grouping of the putative Cprp/DDR48 homologs is not consistent with the known phylogeny in several cases, and the bootstrapping scores are very low. Thus, the tree should not be seen as determining evolutionary relationships between the analysed proteins or fungal species. This may be due to the different proteins not being true homologues, or simply due to the repetitive nature of the protein sequences.

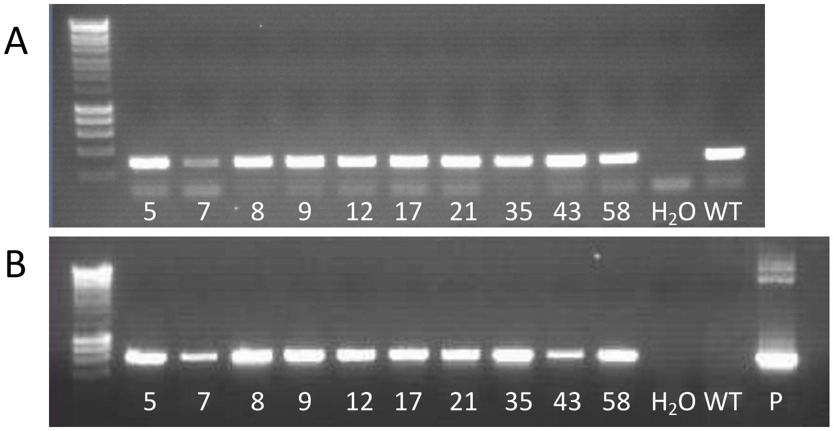

Supplementary Figure 10: PCRs to confirm integration of the *cprp*-AS cassette. A) Positive control for genomic extraction. 282 bp of alpha tubulin amplified using primers CP1-tub-F and CP1-tub-R. Lanes 2 – 11 are cprpAS transformants. WT = wild-type *C. passeckerianus*. B) A 606 bp fragment of the silencing cassette, amplified using primers Agaricus-Gpdprom-F and 456antisense-Int-F. Lanes 2 – 11 are cprpAS transformants. WT = wild-type *C. passeckerianus*. P = plasmid control, using pYES-hph-cprpAS as the template.

1.2 Supplementary Tables

Supplementary Table 1: Two-tailed t tests corresponding to Figure 3D (Pleuromutilin production in transformants containing a cprp antisense silencing construct, as analysed in triplicate by HPLC). Equal variance t tests were performed as all data sets were found to have equal variance according to an f test.

	Cprp1 vs. WT	Cprp2 vs. WT	WT vs. *cprp*AS-8	WT vs. *cprp*AS-12	WT vs. *cprp*AS-17	WT vs. *cprp*AS-21	WT vs. *cprp*AS-35	WT vs. *cprp*AS-43	WT vs. *cprp*AS-58
P value:	4.59E-05	0.000358	0.007284	0.000363	5.12E-06	0.000102	1.78E-06	1.38E-06	9.38E-06

Supplementary Table 2: Two-tailed t tests corresponding to Figure 4B (an assessment of sensitivity to NaCl, analysed in triplicate). Equal variance t tests were performed as all data sets were found to have equal variance according to an f test.

	Y10000 vs. Y16748 (0.3M NaCl)	Y10000 vs. Y16748 (0.5M NaCl)
P value:	0.03187137	0.04629019

Supplementary Table 3: Two-tailed t tests corresponding to Figure 5B (growth rate of yeast strains overexpressing DDR48 or Cprp, analysed in triplicate). Equal variance t tests were performed as all data sets were found to have equal variance according to an f test.

	DDR481 vs WT	DDR482 vs WT	Cprp1 vs WT	Cprp2 vs WT
P value:	2.38037E-05	0.000635081	4.59712E-05	0.000358651

Supplementary Table 4: An analysis of representative DDR48-like proteins present across the higher fungi. Protein composition data and GRAVY (grand average of hydropathicity index) scores were obtained using ExPASy ProtParam (Gasteiger et al., 2005). PI = Protein identity.

Species	Accession number	Protein length	Protein composition					GRAVY	[G/S]Y[G/S] motif				PI with Cprp (%)	PI with DDR48 (%)
			S	N	D	G	Y		SYG	SYS	GYS	GYG		
**Ascomycota**														
**Pezizomycotina**														
Fusarium fujikuroi	XP_023428359.1	243	26.7	21.0	15.6	13.6	9.1	-1.977	18	0	0	0	64.45	55.93
Histoplasma capsulatum	EER43993.1	315	26.0	8.3	13.7	15.6	8.3	-1.813	16	1	0	0	42.54	56.16
Penicillium brasilianum	OOQ87626.1	298	29.1	14.1	13.1	16.4	9.1	-1.750	24	0	0	0	62.28	55.61
Talaromyces verruculosus	KUL83446.1	273	32.2	18.3	13.2	17.2	10.6	-1.866	26	0	0	0	68.50	72.77
Colletotrichum tropicale	KAF4827718.1	305	27.5	13.1	13.1	14.1	8.2	-1.716	21	0	0	1	46.25	60.18
Aspergillus bombycis	XP_022383385.1	260	31.5	13.5	13.8	16.2	11.5	-1.897	23	0	0	1	52.29	51.34
Aspergillus oryzae	EIT81464.1	379	31.4	12.9	10.3	14.0	10.0	-1.519	23	0	0	1	51.16	55.00
**Saccharomycotina**														
Saccharomyces cerevisiae	P18899.4	430	26.5	23.3	13.5	11.4	10.0	-1.960	39	0	0	0	59.55	-
Candida albicans	XP_714253.1	212	31.1	15.1	16.0	13.2	9.9	-1.894	19	0	0	0	75.27	67.06
Candida boidinii	OWB84669.1	311	28.9	22.2	16.4	9.0	9.3	-2.074	23	0	0	0	68.63	60.68
Zygotorulaspora mrakii	QLG71076.1	508	28.1	19.3	13.4	13.0	10.0	-1.844	45	0	0	0	60.00	65.95
Naumovozyma dairenensis	XP_003671904.1	425	23.8	22.1	15.3	10.8	9.9	-1.987	35	1	0	0	52.38	66.39
**Basidiomycota**														
**Agaricomycotina**														
Clitopilus passeckerianus	-	280	23.2	25.0	13.9	11.4	8.2	-2.005	17	1	0	0	-	59.55
Gloeophyllum	XP_007869948.1	246	20.7	17.1	7.7	25.6	6.9	-1.446	11	0	0	2	53.18	48.30
Coprinopsis cinerea	XP_001835064.1	405	24.9	9.9	10.9	13.6	8.4	-1.870	14	2	0	2	47.39	44.40
Heterobasidion irregular	XP_009552756.1	298	21.5	18.8	10.1	22.5	6.4	-1.465	12	0	0	1	46.38	41.49
Trametes versicolor	XP_008040989.1	273	28.6	20.1	10.6	16.5	6.6	-1.659	14	0	0	2	54.55	49.13
Stereum hirsutum	XP_007311260.1	294	23.5	17.3	9.9	20.1	7.1	-1.572	14	0	0	1	52.24	44.20
Punctularia strigosozonata	XP_007388085.1	267	23.2	22.1	9.0	14.2	6.4	-1.565	14	0	0	1	54.66	44.24
**Ustilaginomycotina**														
Testicularia cyperi	PWY96941.1	278	25.5	22.7	11.9	17.3	8.3	-1.871	20	0	0	0	64.00	56.62
Ceraceosorus bombacis	CEH18727.1	249	24.1	17.7	10.4	26.9	6.4	-1.535	14	0	0	0	44.94	45.64
Violaceomyces palustris	PWN49054.1	213	30.0	18.8	11.7	16.0	7.0	-1.762	14	1	0	0	45.78	49.38
**Pucciniomycotina**														
Microbotryum lychnidis-dioicae	KDE05376.1	250	27.6	21.6	8.4	16.0	6.0	-1.550	7	1	1	3	34.12	41.10
Microbotryum silenes-dioicae	SGZ21624.1	253	26.9	22.5	8.3	15.8	6.3	-1.575	6	1	0	2	32.56	40.96
**Blastocladiomycota**														
Allomyces macrogynus	KNE54004.1	270	13.0	1.9	27.0	15.0	27.8	-2.028	15	0	0	13	33.79	40.38
***Homo sapiens* FUS**	sp|P35637|	526	12.9	4.6	4.9	28.9	6.8	-1.319	7	3	2	9	23.91	31.72
***Homo sapiens* TAF15**	sp|Q92804|	592	10.5	4.4	9.3	29.6	9.1	-1.522	4	4	3	23	31.85	33.50

1.3 Supplementary Data

Draft genome sequence data for the *cprp* locus of *C. passeckerianus*. Note that this is data from the diploid strain *C. passeckerianus* DSMZ1602 and the assembly does not differentiate between different alleles, hence the presence of IUPAC code nucleotides. This sequence has been uploaded to GenBank; accession number MW509765.

```
LOCUS       Cprp_locus              3134 bp    DNA     linear
SOURCE      Clitopilus passeckerianus
  ORGANISM  Clitopilus passeckerianus
            Eukaryota; Fungi; Dikarya; Basidiomycota; Agaricomycotina;
            Agaricomycetes; Agaricomycetidae; Agaricales; Entolomataceae;
            Clitopilus.
REFERENCE   1  (bases 1 to 3134)
  AUTHORS   de Mattos-Shipley,K.M.J., Foster,G.D. and Bailey,A.M.
  TITLE     Cprp - An unusual, repetitive protein which impacts pleuromutilin
            biosynthesis in the basidiomycete Clitopilus passeckerianus
  JOURNAL   unpublished
REFERENCE   2  (bases 1 to 3134)
  AUTHORS   de Mattos-Shipley,K.M.J., Foster,G.D. and Bailey,A.M.
  TITLE     Direct Submission
  JOURNAL   Submitted (15-JAN-2021) Life Sciences, University of Bristol, Life
            Sciences Building, Bristol, UK BS8 1TQ, United Kingdom
FEATURES             Location/Qualifiers
     source          1..3134
                     /organism="Clitopilus passeckerianus"
                     /mol_type="genomic DNA"
                     /db_xref="taxon:648682"
     gene            111..815
                     /gene="3-hydroxyanthranilic acid dioxygenase"
     CDS             join(111..275,342..444,503..579,639..815)
                     /gene="3-hydroxyanthranilic acid dioxygenase"
                     /codon_start=1
                     /product="3-hydroxyanthranilic acid dioxygenase"
                     /translation="MPLGPPLNFKKWLSENENLLQPPVNNFCLYKGGDFIVMAVGGPN
                     QRNDYHVNQTEEWFYQHKGGMLLRVVDDDVFRDIRIEEGEMFLLPENTPHNPVRYADT
                     IGLVVERVRPADSDRLRWYCQSGEHKEPTIIYEESFHVTDLGTQLKLVIQRWMLNEDL
                     RKCKACGKVADPK"
     gene            1809..3028
                     /gene="cprp"
     mRNA            join(1809..1942,2004..2691,2748..2814,2870..3028)
                     /gene="cprp"
                     /label=cprp_mRNA
     CDS             join(1866..1942,2004..2691,2748..2814,2870..2880)
                     /gene="cprp"
                     /codon_start=1
                     /product="cprp"
                     /translation="MSYNRDNDNDSYGSGNNDSYGSRKNNDNNNTGGFNDNDNDSYGS
                     KNKRDNNNDSYGSSNNDSYGSSNRRKDDDNDNSNSYGSNNNDNDNSYGSKTNKSNDSY
                     GSSNNDSYGSSNNDSYGSSNRRDNDNDNSYGSKNKDNDSYGSSNNDSYGSKKSDNYGS
                     SNNDTYGSSNRNDNNDSYGSSNKNKDSNDSSYSSSNNDSYGSSNNSSNNDSYGSSNTS
                     SYGSGNNNNNNNNTSSNQSDWVDKGVSYASKQAGYNLDEATADKIGDGLREGFKKFGG
                     GFGN"
BASE COUNT      821 a    793 c    661 g    762 t     97 others
ORIGIN
        1 tttggaaagc gcgttatcaa tgacaacact agatgacggc atcccatgtg cagcatctcc
       61 aaacgccgtc gagtcagtct cttcgtctct tgctctgaag aatagctact atgcctctgg
      121 gacccctct taacttcaag aagtggcttt ctgagaatga aaatcttttg cagcctccag
      181 tcaacaattt ctgtctctac aaaggaggtg acttcatcgt gatggctgtg ggcggcccta
      241 atcaaaggaa cgattaccat gtcaatcaga cagaggtcag cagtgcaatt ggcagccgca
      301 acttcaaagg gaactcaata tctggtcttt gactgaaata ggaatggttc tatcaacaca
      361 aaggcgggat gctcctaagg gttgtggacg acgatgtatt cagagatatc cgaattgaag
      421 agggcgagat gtttttgcta cctggtagat tcatcgtgtc cttgacgcct tagaagctgt
      481 gctcatctcc ctcgctcggt agaaaatacc cctcataacc cggttcggta cgcggatacc
      541 atcgggcttg tggtcgagag agtcaggccc gcagattctg taggcgagta ctcagaacat
      601 tcttgatctc aggagttgct catacgcagt acttgacaga tcgactgcgc tggtactgtc
```

661 agtcaggaga gcataaagaa ccaacaatta tatacgaaga gtcattccac gtcacagacc
721 tcggtactca gctgaagctg gtcattcaga ggtggatgtt gaacgaagac cttcgcaaat
781 gcaaggcttg tggcaaggtg gcagatccga agtaatcaca cctgggccca tgttytyatt
841 tctatcctta ttttytgtta tcccaacata gaaamgtcct scayccgtat acgttcracc
901 gaaactcccy aagcttccaa gacgcaatct arcgcgagat tgwtccaaat ctgcgatgac
961 aattccgccg ccracacacg ttctctgaaa tcggtgtgtt gcgttcggga ctgtcccgcg
1021 atgcaacccc cttcctaggg ggtcagctgg tcaactcaat gttccaagac gggttagtct
1081 ttcaaggcta tcccgccact atctctgttg gcagrtattt tcaggyaagg atgtyrcatc
1141 ctgtcggtaa atgccaaaaa tttgagaaat aagcgctgtc ccgatactgc ggcttcccrg
1201 mtkcacratt arwcgtygag aaawtttytg attctrttcr crggatctct cctttcacyg
1261 cctcgayggg gcgcrckatt ctgtytctct atttcgtggt ttgaacggac ctcggggaac
1321 gggatcatyt gtgccgtaga tcgcttaakm agackatcac actgccacga mtatttgatc
1381 ggtccaaccc aggcctgcct cyytttctcy crtctttcac atccaaaatt ctgcytttgc
1441 kcgtcttttg cyayatatct tgktcggctc actagcatct rctycmtcta ggctgctaaa
1501 ctsmtatcag gctwagtacg ggcacsrggy tcttgcagac aratctggac tccgcygact
1561 ggcacagcaa ttttagcagr tgtcctgsaa tagaacaaaa caaatcttcc attcatsctg
1621 tcatttscaa ggccccagtt ccacattggt ccatcatcta tttcattacg catcagggcr
1681 atgatggrat atgttcgctc ctgaaatggc gacattacga agcggrywgc cggratctct
1741 gagyagggtc tgggtctggg gagatataaa atgggttgaa ccgatcttgr ttgaagcatc
1801 gtctcttcgg actctcagat cctgaacaac tagctcttgc atctctatcg tatctcttct
1861 tagctatgtc ttacaaccgc gacaacgata acgattccta tggctccggc aacaacgaca
1921 gctacggcag tcgtaagaac aagtacgytc mtctctrcak gtkgatatct cgcrgacgtt
1981 ctgatactkk atttctcygt cagtgacaac aacaacacgg gaggcttcaa tgacaacgac
2041 aacgactcct acggttccaa gaacaagcgt gataacaaca acgattccta cggttccagc
2101 aacaacgact cctatggatc gtctaaccgc cgcaaggacg acgacaatga taactcgaac
2161 tcctacggct ctaacaacaa cgacaatgat aactcgtatg gctcaaagac gaacaagagc
2221 aacgactcgt acggctcatc caacaacgat tcgtatggat cgtccaacaa tgattcttat
2281 ggctcgtcca accgccgcga taacgacaac gacaatagct atggttccaa gaacaaggat
2341 aacgatagct acggttcttc caacaacgac agctacggtt ccaagaagag cgacaactat
2401 ggttcctcca acaacgacac ttacggctcc tccaaccgta acgacaacaa cgatagctat
2461 ggctcttcta acaagaacaa ggacagcaac gattccagct acagctcttc caacaacgat
2521 agctacggtt cctccaataa cagcagcaac aatgatagct atggctcgag caacacctcg
2581 tcttatggat ctggcaacaa caataacaac aacaacaaca cctcctcgaa ccagagtgac
2641 tgggtcgaca agggcgtctc gtatgcctct aagcaagcgg gttacaacct cgtgagtaty
2701 kccagcagcg tactytgtat actatrtctc atrcaaactc yctttaggat gaggcaactg
2761 ccgacaagat cggcgatggt ctcagggagg gcttcaagaa gttcggagga ggatgtragt
2821 tcttsaytcg tttcgcyyca acctttctta ataacctctc atctttcagt cggaaactaa
2881 gggcactcgg gtgaatggag cgcttaacat cggaatggac actgtaaaat atggaattag
2941 gaattattct cgcatttctt gataacaaat gttgtatttc ggcttgtata ttctcgtaaa
3001 ctacaaatca agaattcgta ttcgaactrr cgtktrccac tcatgaggar gmcgggccgt
3061 tcctccaaac agtctattya gccctccccc ttccatgcct tgcatgctca ggckaaagcg
3121 aacagtgcca ccaa

1.4 References

Gasteiger E, Hoogland C, Gattiker A, Wilkins MR, Appel RD, Bairoch A, 2005. Protein identification and analysis tools on the ExPASy server. In. *The proteomics protocols handbook.* Springer, 571-607.
Kumar S, Stecher G, Li M, Knyaz C, Tamura K, 2018. MEGA X: molecular evolutionary genetics analysis across computing platforms. *Molecular Biology and Evolution* **35**, 1547-9.
Lancaster AK, Nutter-Upham A, Lindquist S, King OD, 2014. PLAAC: a web and command-line application to identify proteins with prion-like amino acid composition. *Bioinformatics* **30**, 2501-2.
Pieuchot L, Lai J, Loh RA*, et al.*, 2015. Cellular subcompartments through cytoplasmic streaming. *Developmental cell* **34**, 410-20.
Sievers F, Higgins DG, 2018. Clustal Omega for making accurate alignments of many protein sequences. *Protein Science* **27**, 135-45.
Teichert S, Schönig B, Richter S, Tudzynski B, 2004. Deletion of the Gibberella fujikuroi glutamine synthetase gene has significant impact on transcriptional control of primary and secondary metabolism. *Mol Microbiol* **53**, 1661-75.
Wiemann P, Sieber CM, Von Bargen KW*, et al.*, 2013. Deciphering the cryptic genome: genome-wide analyses of the rice pathogen Fusarium fujikuroi reveal complex regulation of secondary metabolism and novel metabolites. *PLoS Pathog* **9**, e1003475.
